# Cancer cell autophagy, reprogrammed macrophages, and remodeled vasculature in glioblastoma triggers tumor immunity

**DOI:** 10.1016/j.ccell.2022.08.014

**Published:** 2022-10-10

**Authors:** Agnieszka Chryplewicz, Julie Scotton, Mélanie Tichet, Anoek Zomer, Ksenya Shchors, Johanna A. Joyce, Krisztian Homicsko, Douglas Hanahan

**Affiliations:** 1 Swiss Institute for Experimental Cancer Research (ISREC), School of Life Sciences, Swiss Federal Institute of Technology Lausanne (EPFL), Lausanne, Switzerland; 2 Agora Translational Cancer Research Center, Lausanne, Switzerland; 3 Lausanne Branch, Ludwig Institute for Cancer Research, Lausanne, Switzerland; 4 Department of Oncology, University of Lausanne, Lausanne, Switzerland; 5 Swiss Cancer Center Leman (SCCL), Lausanne/Geneva, Switzerland

**Keywords:** glioblastoma immunotherapy, repurposing tricyclic antidepressants, immunostimulatory autophagy, reprogramming immunosuppressive macrophages, histamine receptor signaling, remodeling tumor vasculature, VEGF inhibitors, high endothelial venules, anti-PD-L1 immune checkpoint blockade, multi-targeted cancer therapy

## Abstract

Glioblastoma (GBM) is poorly responsive to therapy and invariably lethal. One conceivable strategy to circumvent this intractability is to co-target distinctive mechanistic components of the disease, aiming to concomitantly disrupt multiple capabilities required for tumor progression and therapeutic resistance. We assessed this concept by combining vascular endothelial growth factor (VEGF) pathway inhibitors that remodel the tumor vasculature with the tricyclic antidepressant imipramine, which enhances autophagy in GBM cancer cells and unexpectedly reprograms immunosuppressive tumor-associated macrophages via inhibition of histamine receptor signaling to become immunostimulatory. While neither drug is efficacious as monotherapy, the combination of imipramine with VEGF pathway inhibitors orchestrates the infiltration and activation of CD8 and CD4 T cells, producing significant therapeutic benefit in several GBM mouse models. Inclusion up front of immune-checkpoint blockade with anti-programmed death-ligand 1 (PD-L1) in eventually relapsing tumors markedly extends survival benefit. The results illustrate the potential of mechanism-guided therapeutic co-targeting of disparate biological vulnerabilities in the tumor microenvironment.

## Highlights

• Combining imipramine (IM) with VEGF pathway inhibitors impairs glioma progression
• IM + anti-VEGF increases autophagic flux and remodels vasculature to promote immunity
• IM reprograms immunosuppressive TAMs by inhibiting histamine receptor signaling
• IM + anti-VEGF therapy sensitizes gliomas to anti-PD-L1 so as to sustain immunity

## Graphical abstract

**Figure:**
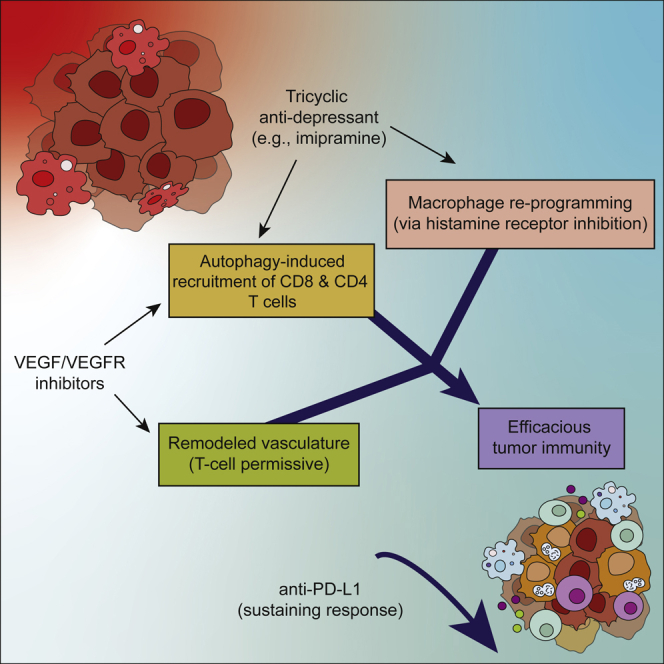

## Teaser

Chryplewicz et al. describe effects of a tricyclic antidepressant and VEGF inhibitors in glioblastoma. Impramine increases autophagic flux in cancer cells, which recruits T cells, and reprograms macrophages as pro-inflammatory by inhibiting histamine receptor. VEGF inhibitors remodel tumor vasculature, enabling T cell invasion and activation. The result is immune-dependent therapeutic efficacy, augmentable by anti-PD-L1.

## Introduction

Glioblastoma (GBM) is an invasive form of brain cancer with a robust vasculature arising via angiogenesis and co-option of normal blood vessels (Ostrom et al., 2018; Xie et al., 2014). Radiotherapy (RT) combined with chemotherapy (temozolomide [TMZ]) is the standard of care, but the relapse-free state is transitory (15 months on average) (Stupp et al., 2005, 2009). The vascular endothelial growth factor (VEGF) blocking antibody bevacizumab is approved, despite a lack of survival benefit, largely due to its effects in reducing edema (Chinot et al., 2014; Gilbert et al., 2014; Li et al., 2017). Other therapeutic agents have been tested, largely to no avail (Pearson and Regad, 2017; Quail and Joyce, 2017; Reardon et al., 2017; Schalper et al., 2019). As such, new treatment strategies are needed.

We have previously reported, using a genetically engineered mouse model of GBM, that two generic drugs can be re-purposed as a novel therapeutic strategy for GBM. The combination of a tricyclic antidepressant (TCA) and an anti-coagulant of a class that inhibits the P2RY_12_ receptor hyper-activate already elevated levels of autophagy in gliomas (Shchors et al., 2015). Their combinatorial benefit is ascribed to concerted elevation autophagy. Imipramine (IM, a TCA) and ticlopidine (TIC, a P2RY_12_ inhibitor) produces a significant yet limited survival benefit in glioma-bearing mice. We envisaged that co-targeting distinctive tumor-promoting mechanisms along with autophagy could produce added benefit. We focused on the angiogenic tumor vasculature, reasoning that VEGF pathway inhibitors might have benefit if so combined.

The results presented below reveal remarkable synergies upon combining a re-purposed TCA (imipramine) with drugs that inhibit VEGF-VEGF receptor (VEGFR) signaling.

## Results

**Figure 1:**
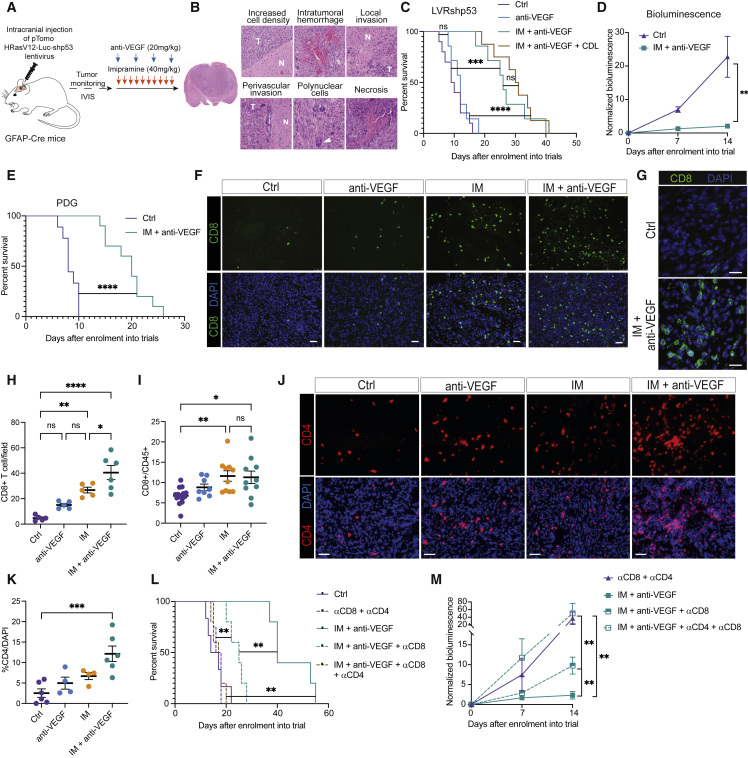
IM + anti-VEGF prolongs survival of GBM mice and is immunostimulatory (A) Schematic of the long-term therapeutic trials in the lentiviral-induced mouse model of glioma. (B) Representative images of H&E-stained tissue sections from a tumor that developed in an end-stage LVRshp53 animal. Scale bar, 30 μm. Representative of whole-slide images of three tumors. (C) Survival of tumor-bearing LVRshp53 animals subjected to the indicated treatments. Control (Ctrl) (n = 10), anti-VEGF (n = 7), IM + anti-VEGF (n = 7), IM + anti-VEGF + CDL (n = 8). (D) Normalized bioluminescence in LVRshp53 animals treated as indicated for 2 weeks. (E) Survival of PDG animals subjected to the indicated treatments. Ctrl (n = 9), IM + anti-VEGF (n = 10). (F) Representative images of CD8 (green) and DAPI nuclear staining (blue). Scale bar, 50 μm. Image is illustrative of the analysis shown in (H). (G) High-magnification images of CD8 T cells in a Ctrl versus an IM + anti-VEGF-treated tumor. Scale bar, 50 mm (H) Quantification of CD8 T cells in LVRshp53 tumors treated as indicated for 12 days. Each dot indicates the average of 8–12 immuno-stained tumor tissue sections from one mouse. (I) Flow cytometry analysis of CD8 T cells in LVRshp53 tumors treated as indicated for 12 days. Cells were gated as CD45^+^CD3^+^CD8^+^. Ctrl (n = 15), anti-VEGF (n = 8), IM (n = 10), IM + anti-VEGF (n = 10). (J and K) Representative images (J) and quantification (K) of CD4 T cells in whole LVRshp53 tumor tissue section. Animals were treated for 12 days. Scale bar, 50 μm. Ctrl (n = 6), anti-VEGF (n = 4), IM (n = 4), IM + anti-VEGF (n = 6). (L) Assessment of the functional contributions of CD8 and CD4 T cells to survival benefit. Ctrl (n = 6), αCD8 + αCD4 (n = 4), IM + anti-VEGF (n = 5), IM + anti-VEGF + αCD8 + αCD4 (n = 5), IM + anti-VEGF + αCD8 (n = 5). (M) Normalized bioluminescence in LVRshp53 mice treated as indicated in (L). (Para break) Data in all quantitative panels are shown as mean ± SEM. ^∗^p < 0.05; ^∗∗^p < 0.01; ^∗∗∗^p < 0.001; ^∗∗∗∗^p < 0.0001; ns, no statistical significance. For survival analyses, Mantel-Cox test was performed. Other analyses by Mann-Whitney or one-way ANOVA tests.

The aforementioned study (Shchors et al., 2015) was largely conducted using two genetically engineered mouse models (GEMMs) that differ in suffering heterozygous (GRLp53flhet) or homozygous (GRLp53flko) deletions of the p53 tumor suppressor gene (Figure S1A). While informative, these models have proved cumbersome for pre-clinical trials due to multifocal and temporally variable neoplastic progression. Therefore, we established a mouse model of gliomagenesis (Friedmann-Morvinski et al., 2012; Marumoto et al., 2009) involving stereotactic inoculation into the hippocampus of a lentivirus conditionally expressing an activated HRasV12 oncogene and an shRNA that knocks down expression of the p53 tumor suppressor, along with a luciferase reporter (LVRshp53; Figures 1A and S1B). The recipient mice harbor a GFAP-Cre transgene that activates expression of the lentiviral delivered HRasV12 and luciferase (Friedmann-Morvinski et al., 2012; Marumoto et al., 2009). While Ras genes are infrequently mutated in human GBM (∼2%; Prior et al., 2020), the RAS/MAPK pathway is frequently activated via mutational loss of the NF1 tumor suppressor gene (14%–23% of GBM; Philpott et al., 2017). A comparative analysis of mouse GBM elicited by lentiviruses delivering shNF1 versus HRasV12, each along with shp53, revealed similar molecular and pathologic phenotypes (Friedmann-Morvinski et al., 2012). Notably, both of these models (and the aforementioned transgenic models) phenocopy the mesenchymal subtype of human GBM, which is associated with worse outcome and response to therapy (Phillips et al., 2006). Moreover, the HrasV12 shp53 lentivirus-based model has been profiled via single-cell RNA sequencing and shown to be comparable with human GBMs in regard to cellular plasticity, wherein a single glioma can present with multiple cellular states and putative molecular subtypes in varying abundances (Neftel et al., 2019). Tumors can be monitored non-invasively (Figure S1C), and present with histological features of human GBM (Figure 1B), including high rates of proliferation (Figure S1D) and aberrant vasculature (Figure S1E).

### Assessing the combination of the TCA imipramine and VEGF/VEGFR inhibitors

An initial pilot study investigated the therapeutic efficacy of two VEGFR TKIs, sunitinib and axitinib, alone and in combination with imipramine in the GRLp53flhet transgenic mouse model. Consistent with clinical results (Duerinck et al., 2018; Hutterer et al., 2014), monotherapy with the VEGFR inhibitors had minimal effect: neither improved overall survival (Figure S1F). Interestingly, however, both drugs further enhanced the survival benefit of IM compared with IM monotherapy (Figure S1F) and were well tolerated. Since the anti-VEGF antibody bevacizumab is approved for clinical use in GBM, whereas no VEGFR TKIs are approved, we switched to using an analog of bevacizumab, B20S, that binds to and sequesters mouse VEGF.

Much as for the clinical experience, the anti-VEGF antibody had no survival benefit as monotherapy in the GRLp53flko (Figure S1G) or LVRshp53 (Figure 1C) models. However, as for the VEGFR TKIs, B20S similarly enhanced the therapeutic efficacy of IM with or without inclusion of the P2RY_12_ inhibitors ticlopidine (TIC) in the GRLp53flko model (Figure S1G) or clopidogrel (CDL) in the LVRshp53 model (Figure 1C). The combinatorial treatment of IM + anti-VEGF significantly delayed tumor growth (Figure 1D). In addition, both double and triple combinations of anti-VEGF and IM ± CDL reduced tumor burden (Figure S1H). Motivated by the initial results that VEGFR TKIs also showed combinatorial benefit with IM (Figure S1F), and by the consideration that oral TKIs might in some cases be preferential for patients to the intravenously (i.v.) dosed monoclonal anti-VEGF antibody, we further evaluated axitinib, which is clinically approved for other indications (Motzer et al., 2013). Our results indicate that axitinib has similar survival benefit to anti-VEGF when combined with IM or IM + CDL in the LVRshp53 model (Figures S1I–S1K).

We next asked if the combo treatments were effective in other immunocompetent pre-clinical mouse models of GBM. We first assessed the genetically engineered platelet-derived growth factor-driven (PDG) model of *de novo* gliomagenesis glioma (Hambardzumyan et al., 2016; Pyonteck et al., 2013). IM + anti-VEGF also delayed tumor progression and increased survival benefit in the proneural PDG model (Figures 1E, S1L, and S1M). We also performed trials in the syngeneic GL261 orthotopic transplant model (Szatmári et al., 2006), revealing discernible efficacy for IM as monotherapy, which was enhanced by anti-VEGF (Figure S1N).

We next assessed the levels of autophagy when anti-VEGF was included in the various therapeutic regimens. Interestingly, anti-VEGF alone modestly enhanced autophagy as revealed by co-localization of LC3 and LAMP1, which was further elevated in combinations with IM ± CDL (Figures S2A and S2B). Since IM + TIC elevated levels of autophagy by coordinately increasing the levels of cAMP in gliomas (Shchors et al., 2015), we analyzed combinations with anti-VEGF. Indeed, IM + anti-VEGF elevated cAMP concentrations compared with controls (Figure S2C). Notably, inclusion of the P2RY_12_ inhibitor did not further elevate cAMP levels, consistent with its inability to improve upon the survival benefit of IM + anti-VEGF.

### CD8 and CD4 T cells contribute to therapeutic efficacy

Intrigued by reports that autophagy in tumors could be immunogenic (Ladoire et al., 2016a, 2016b; Michaud et al., 2011; Pietrocola et al., 2016), we investigated the possibility that IM ± anti-VEGF therapy might be attracting CD8 T cells, by analyzing tumors from mono- and combination therapy cohorts in LVRshp53 and PDG GBM models. While CD8 T cells were rare in the untreated and anti-VEGF-treated tumors, increased numbers were observed in tumors treated with IM alone, and markedly elevated in the combinatorial arm (Figures 1F–1I, S2D, and S2E). Furthermore, we performed a similar analysis involving axitinib and observed a significant enhancement of CD8 T cell accumulation when combined with IM (Figures S2F and S2G). To begin assessing the possibility that imipramine could have a similar immunostimulatory effect in other tumor types, we treated the iBIP2 GEMM of BRAF-driven melanoma (Figure S2H). Monotherapy with imipramine reduced tumor burden (Figure S2I), and similarly induced CD8 T cell infiltration (Figures S2J–S2L).

To ascertain whether induction of CD8 T cell infiltration was connected to increased levels of autophagic flux upon IM + anti-VEGF treatment, we silenced the expression of ATG3, a key regulator of autophagy, in glioma-derived cells, which were implanted into the brains of immunocompetent animals (Figures S2M and S2N). Mice bearing shATG3 gliomas survived similarly to those with control tumors (Figure S2O); indicating that the intrinsic level of autophagy in gliomas was not modulating tumor progression, in contrast to other tumor types (Chavez-Dominguez et al., 2020). However, the survival benefit of mice treated with the IM + anti-VEGF was abrogated in shATG3 tumors (Figure S2O) and associated with reduced CD8 T cell infiltration, in comparison with similarly treated control (ATG3-proficient) tumors (Figures S2P and S2Q), revealing the importance of autophagic flux in CD8 T cell recruitment.

Given the multifaceted roles of CD4 T cells in anti-tumor immune responses (Alspach et al., 2019; Spitzer et al., 2017; Zander et al., 2019), we assessed their presence following the combo therapy. The abundance of CD4 T cells was significantly expanded in double-treated tumors in the LVRshp53 and PDG models (Figures 1I–1K, S2R, and S2S).

We next assessed the functional contributions of CD8 and CD4 T cells to therapeutic efficacy by implanting glioma-derived cells in parallel into syngeneic and immunocompromised mice. Notably, there was no therapeutic response in immunodeficient mice (Figure S2T) compared with syngeneic immunocompetent mice (Figure S2U), emphasizing the role of the adaptive immune system in driving the responses to this regimen. In addition, we included depleting αCD8 ± α-CD4 antibodies in IM + anti-VEGF treated LVRshp53 cohorts. Concomitant depletion of CD4 and CD8 T cells (Figure S2V) led to a complete abrogation of the therapeutic benefit of IM + anti-VEGF (Figures 1L and 1M), implicating both T cell subtypes in therapeutic efficacy. In addition, we depleted T cells in tumors treated with our previously reported (Shchors et al., 2015) autophagy-inducing combination of IM and a P2RY_12_ inhibitor, which also negated therapeutic efficacy (Figure S2W), further linking the therapeutic benefit of imipramine with autophagy-dependent recruitment of CD8 and CD4 T cells.

**Figure 2:**
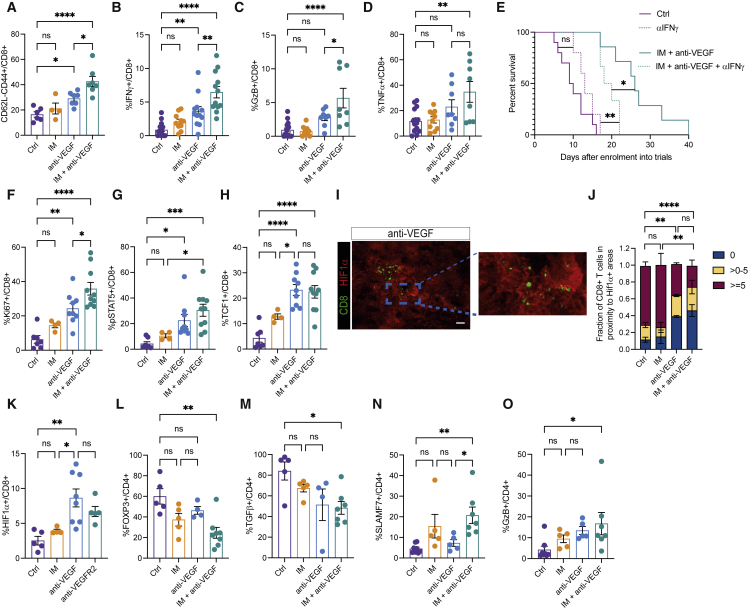
CD8 and CD4 T cells are activated upon IM + anti-VEGF treatment (A) Flow cytometry analysis of effector T cells (CD62L-CD44^+^). Ctrl (n = 6), IM (n = 4), anti-VEGF (n = 7), and IM + anti-VEGF (n = 7). (B) FACS analysis of IFNγ intracellular staining in fixed and permeabilized CD8+T cells. Ctrl (n = 16), IM (n = 10), anti-VEGF (n = 12), IM + anti-VEGF (n = 12). (C and D) Flow cytometry analysis of GzB (C) and TNFα (D) intracellular staining in CD8 T cells. Ctrl (n = 15), IM (n = 10), anti-VEGF (n = 8), IM + anti-VEGF (n = 8). (E) Functional importance of IFNγ for the survival of LVRshp53 animals subjected to the indicated treatments. Ctrl (n = 9), anti-IFNγ (n = 5), IM + anti-VEGF (n = 7), IM + anti-VEGF + anti-IFNγ (n = 6). Statistical analysis by Mantel-Cox test. (F, G, and H) Flow cytometry analysis of Ki67 (F), pSTAT5 (G), and TCF1 (H) in CD8 T cells. Ctrl (n = 7), IM (n = 4), anti-VEGF (n = 9), and IM + anti-VEGF (n = 10). (I) Representative image of HIF-1α (red) and CD8 (green) in the anti-VEGF-treated tumor. Image is illustrative of the analysis performed in (J). Scale bar, 50 mm (J) Quantification of the proximity of CD8 T cells to hypoxic regions in the entire area of full sections of GBM tumors. The zones were divided into 0 μm (i.e., within the HIF-1α+ zone), >0 and <5 μm, and >5 μm separating T cells and HIF-1α+ regions. Ctrl (n = 11), IM (n = 4), anti-VEGF (n = 4), IM + anti-VEGF (n = 5). (K) Flow cytometry analysis of intracellular HIF-1α expression in fixed and permeabilized CD8 T cells. Ctrl (n = 5), IM (n = 4), anti-VEGF (n = 8), anti-VEGFR2 (n = 5). (L) Flow cytometry analysis of intracellular FOXP3 expression in CD4 T cells. Ctrl (n = 5), IM (n = 5), anti-VEGF (n = 4), IM + anti-VEGF (n = 8). (M) Flow cytometry analysis of intracellular TGFβ expression in CD4 T cells. Ctrl (n = 5), IM (n = 5), anti-VEGF (n = 4), IM + anti-VEGF (n = 7). (N and O) Flow cytometry analysis of SLAMF7 (N) and GzB (O) expression in CD4 T cells. Ctrl (n = 8), IM (n = 5), anti-VEGF (n = 5), IM + anti-VEGF (n = 7). (Para break) Data in all quantitative panels are shown as mean ± SEM. ^∗^p < 0.05; ^∗∗^p < 0.01; ^∗∗∗^p < 0.001; ^∗∗∗∗^p < 0.0001; ns, no statistical significance. Statistical analysis by one-way ANOVA, unless otherwise indicated.

We next characterized the CD8 T cells populating GBM tumors, and found a modest increase in CD62L-CD44^+^ effector cells in anti-VEGF-treated tumors, which were significantly expanded upon dual therapy compared with untreated and IM-monotherapy arms (Figure 2A). The activation marker interferon gamma (IFNγ) was significantly increased in the combo arm, as well as in the anti-VEGF-alone arm (Figure 2B). TNFα and Granzyme B (GzB) were also expressed at higher levels in IM + anti-VEGF-treated tumors and trended toward higher levels in the anti-VEGF-alone arm, compared with untreated or IM-treated tumors (Figures 2C and 2D). Similar to the mesenchymal GBM model, these markers were also elevated in proneural tumors treated with the IM + anti-VEGF regimen compared with controls and with monotherapy with IM (Figures S3A–S3C).

To assess the functional contribution of IFNγ expression in CD8 T cells to the efficacy of the IM + anti-VEGF combo, we included an IFNγ-blocking antibody in the therapeutic regimen. Blockade of IFNγ significantly reversed therapeutic efficacy (Figure 2E), establishing that activation of IFNγ signaling in CD8 T cells was integral to the anti-tumoral responses evoked by IM + anti-VEGF.

In addition, the proliferative phenotype of CD8 T cells was increased in tumors treated with the double combination, as assessed by Ki67 expression and STAT5 phosphorylation (Figures 2F and 2G). Recently, TCF1 has been described as a marker of a stem-cell-like subset of CD8 T cells associated with improved anti-tumor immunity and response to immune-checkpoint blockade (Sade-Feldman et al., 2018; Siddiqui et al., 2019). We found that CD8 T cells were highly expressing TCF1 upon IM + anti-VEGF therapy (Figure 2H). Interestingly, the VEGF inhibitory component of the combo regimen was primarily responsible for the induction of proliferative and stem-like CD8 T cells (Figures 2F–2H). Guided by reports that anti-VEGF therapy increases hypoxia within viable tumor areas (Franco et al., 2006; Shi et al., 2017) and that low oxygen bioavailability enhances the cytotoxic function of CD8 T cells (de Almeida et al., 2020; Doedens et al., 2013), we found increased tumor hypoxia and accumulation of CD8 T cells within hypoxic regions (Figures 2I and 2J). Moreover, CD8 T cells from the anti-VEGF arm expressed higher levels of the hypoxia-inducible factor HIF-1α (Figure 2K). The results are congruent with a report that hypoxia activates effector functions of CTLs (de Almeida et al., 2020; Xu et al., 2016).

Given that CTLs can trigger apoptosis, combo-treated tumors treated were assessed by immunostaining for cleaved caspase 3 (CC3), which is diagnostic of ongoing apoptosis. The cohorts treated with IM + anti-VEGF evidenced elevated levels of CC3 (Figures S3D and S3E). The observed apoptosis was CD8 dependent, as the levels of CC3 were reduced when aCD8 was used to deplete CTLs in these cohorts (Figures S3F and S3G).

Since CD4 T cells are highly versatile and play important roles in coordinating immune responses, we assessed their functions. We found that CD4 T cells present in the untreated tumors were of the regulatory (Treg) phenotype that expressed transforming growth factor beta (TGFβ), a cytokine broadly implicated in immunosuppression (Figures 2L and 2M). Treatment with IM + anti-VEGF decreased intra-tumoral Tregs and levels of TGFβ (Figures 2L and 2M). We next performed a short-term *in vivo* depletion of CD4 T cells to assess conventional T-helper function, which revealed decreased CD8 T cell numbers concomitant with reduced expression of IFNγ and GzB (Figures S3H–S3J). Noting that CD4 T cells are also capable of direct cytotoxicity against tumor cells (Oh et al., 2020, p. 4; Quezada et al., 2010), we assessed markers linked to cytotoxic function in CD4 T cells (Cachot et al., 2021), revealing higher expression of SLAMF7 and GzB within this population recruited by the dual combinatorial therapy, indicative of their cytotoxic potential (Figures 2N and 2O).

In sum, the results indicate that the combination of IM + anti-VEGF is immunostimulatory and that both CD8 and CD4 T cells are cooperatively contributing to therapeutically effective immune responses in mouse models of GBM.

### Tumor vascularity is reduced and remodeled in the IM + anti-VEGF-based combinations

**Figure 3:**
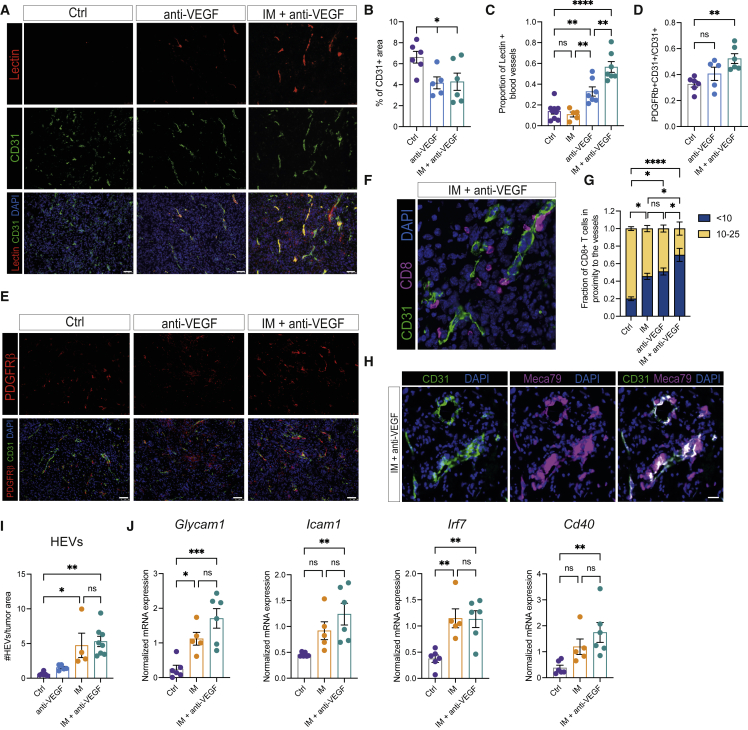
Anti-VEGF alone and in combination with IM remodels the glioblastoma tumor vasculature (A) Representative images of systemically perfused lectin (red), CD31 (green), and DAPI (blue) of LVRshp53 tumors treated as indicated for 1 week. Images are illustrative of the analysis shown in (B) and (C). Scale bar, 50 mm (B) Percentage of CD31^+^ area in LVRshp53 tumors treated as indicated for 1 week. Each dot indicates the average of 8–12 fields in tissue sections from a GBM tumor from one mouse. Ctrl (n = 6 tumors), anti-VEGF (n = 5), IM + anti-VEGF (n = 6). (C) Proportion of i.v.-infused lectin and CD31 co-localization as a percentage of CD31^+^ area. Each dot indicates the average of 8–12 fields in tissue sections from a GBM tumor from one mouse (n = 5–6 tumors for each group). (D and E) Quantification (D) and representative images (E) of PDGFRβ and CD31 co-localization as a percentage of CD31-positive area. Each dot indicates the average of 8–12 fields in tissue sections from a GBM tumor (n = 5–6 tumors for each group). Scale bar, 50 μm. (F and G) Representative image (F) and quantification (G) of a fraction of CD8^+^ T cells located within 10 μm or beyond (10–25 μm) the closest CD31^+^ blood vessel in the entire area of the GBM tumor section. Ctrl (n = 6), IM (n = 4), anti-VEGF (n = 6) and IM + anti-VEGF (n = 6). Scale bar, 50 μm. (H and I) Representative immunofluorescence (H) of MECA79 (magenta), CD31 (green), and DAPI (blue) and HEVs quantification (I) in the entire area of a full tumor tissue section. Ctrl (n = 6), anti-VEGF (n = 5), IM (n = 4), IM + anti-VEGF (n = 8). Scale bar, 20 μm. (J) mRNA expression of endothelial cell markers. Data are normalized to 18S RNA. (Para break) Data in all quantitative panels are presented as mean ± SEM. ^∗^p < 0.05; ^∗∗^p < 0.01; ^∗∗∗^p < 0.001; ^∗∗∗∗^p < 0.0001; ns, no statistical significance. Statistical analysis by one-way ANOVA, unless otherwise indicated.

The density and integrity of the angiogenic and morphologically aberrant tumor vasculature in GBM were assessed in cohorts of mice treated with anti-VEGF, IM + anti-VEGF, and IM + anti-VEGF + CDL. Despite a lack of survival benefit, monotherapy with anti-VEGF significantly reduced the density of CD31^+^ blood vessels in tumors; the inclusion of IM had no effect (Figures 3A and 3B), although endothelial cells were more distant from hypoxic areas in tissues treated with the dual therapy (Figure S4A). Vascular functionality and integrity, as measured by fluorescein-labeled lectin perfusion and pericyte coverage, respectively, were improved by anti-VEGF alone and further enhanced with IM (Figures 3C–3E). Addition of CDL did not alter vascular functionality, although it increased coverage by mature pericytes (Figures S4B–S4E) (Song et al., 2005). Such pseudo-normality is associated with an increased capability of CD8 T cells to extravasate into tumors (Allen et al., 2017). We therefore analyzed the spatial distribution of CD8 T cells in GBM tumors and found a subset to be preferentially localized close to the blood vessels in IM-treated tumors. This effect was further enhanced when anti-VEGF was included (Figures 3F and 3G). Notably, T cells also localize to hypoxic regions, as shown in Figure 2J. These data suggest that the processes of T cell intravasation across the functionally remodeled vasculature and hypoxia-dependent T cell activation could constitute spatially distinct stages in the induction of an efficacious anti-tumoral immune response. In addition, we assessed the formation of high endothelial venules (HEVs) on the vasculature, known to promote recruitment of lymphocytes into tissues during inflammatory responses (Sautès-Fridman et al., 2019). We observed MECA79+ vessels displaying the distinctive morphological features of HEVs scattered throughout tumors treated with IM + anti-VEGF in both LVRshp53 and PDG models (Figures 3H, S4F and S4G). Imipramine was the necessary component for induction of HEVs (Figure 3I). Concordantly, immunohistochemical analysis of melanoma tumors treated with IM monotherapy also revealed the presence of HEVs, which we envisage contribute to the similarly robust CD8 T cell influx elicited in this very different tumor type (Figures S4H and S4I). In addition to histological analyses, we isolated CD31^+^ cells from GBM and melanoma tumors using magnetic beads and performed transcriptional profiling. We found that, upon treatment with IM ± anti-VEGF, tumor endothelial cells expressed higher levels of *Glycam1*, which is expressed in mature HEVs (Figures 3J, S4J, and S4K). In addition, we observed increased expression of pro-inflammatory mediators (e.g., *Icam1*, *Cd40*, *Irf7*) in tumor endothelial cells from both mesenchymal and proneural GBM tumors as well as in melanoma (Figures 3J, S4J and S4K), consistent with more efficient T cell trafficking and suggestive of their contributions to potent anti-tumor immunity.

### Imipramine reprograms tumor-associated macrophages

**Figure 4:**
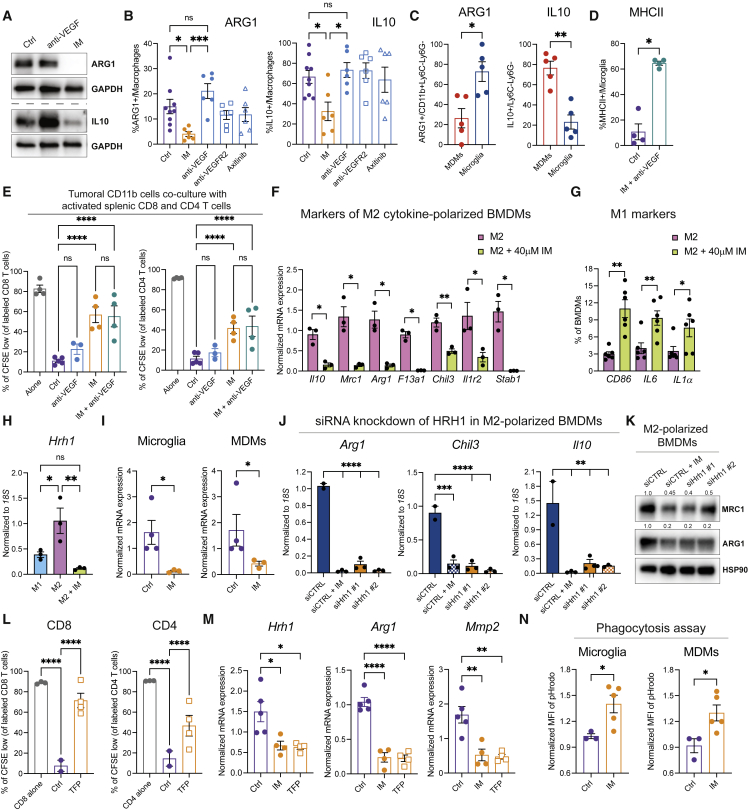
Imipramine downregulates an M2-like program in TAMs (A) Western blot analysis of ARG1 and IL-10 in single tumors treated or not with IM or anti-VEGF. (B) Flow cytometry analysis of ARG1 and IL-10 expression in GBM tumors treated for 1 week. Ctrl (n = 9 tumors), IM (n = 6), anti-VEGF (n = 6), anti-VEGFR2 (n = 6), Axitinib (n = 6). Macrophages were gated as CD45^+^CD11b+Ly6C-Ly6G−. (C) Expression of ARG1 and IL-10 in microglia (CD49d−) and MDMs (CD49d+) assessed by FACS in untreated tumors (n = 5). (D) Expression of MHC-II within microglia as assessed by flow cytometry. Ctrl (n = 4 tumors) and IM + anti-VEGF (n = 4). (E) *Ex vivo* co-cultures of tumoral CD11b cells and activated splenic CFSE-labeled CD8 or CD4 T cells. Each dot represents the average of two or three technical replicates. T cells alone (n = 4), Ctrl co-culture (n = 5), anti-VEGF (n = 3), IM (n = 4), IM + anti-VEGF (n = 4). (F) Analysis of the M2-like program in cytokine-polarized macrophages as assessed by qRT-PCR analysis of Ctrl and IM-treated M2-like BMDMs. Expression is normalized to *18S* statistics by Welch’s t test. Each dot represents an individual sample. Data are representative of three independent experiments. (G) Analysis of the M1-like program in BMDMs assessed by FACS. Each dot represents an individual replicate. (H) Expression of *Hrh1* mRNA normalized to *18S* in *ex vivo* M1-and M2-polarized BMDMs, either untreated or IM treated for 24 h. Each dot represents an individual sample. Data are representative of three independent experiments. (I) mRNA expression of *Hrh1* in FACS-sorted microglia or MDMs from Ctrl and IM-treated tumors. (J) mRNA expression of *Arg1*, *Chil3*, and *Il10* in M2-polarized macrophages that were transfected with si*Ctrl* or two different si*Hrh1* constructs. Cells were treated with 40 μm IM for 24 h. Data are representative of two independent experiments. (K) Western blot analysis of MRC1 and ARG1 expression of siRNA-transfected M2 BMDMs. Data are representative of two independent experiments. (L) CD8 and CD4 T cell proliferation during co-culture with tumoral CD11b cells isolated from untreated (n = 2) or TFP-treated tumors (n = 4). (M) mRNA expression of *Hrh1*, *Arg1*, and *MMP2* in CD11b cells isolated from tumors treated with IM (n = 4), TFP (n = 4), or untreated Ctrl (n = 5). (N) Phagocytosis assay involving sorted microglia and MDMs from untreated or IM-treated tumors assayed with green pHrodo *S. aureus* bioparticles. Data presented as mean fluorescence intensity (MFI) of pHrodo/live cells. (Para break) Data in all quantitative panels are presented as mean ± SEM ^∗^p < 0.05; ^∗∗^p < 0.01; ^∗∗∗^p < 0.001; ^∗∗∗∗^p < 0.0001; ns, no statistical significance. Statistical analysis by Mann-Whitney test or one-way ANOVA, unless otherwise stated.

In GBM, tumor-associated macrophages (TAMs), arising both from monocyte-derived macrophages (MDMs) and resident microglia, are pro-tumorigenic and associated with immunosuppression (Bowman et al., 2016; Hambardzumyan et al., 2016; Hussain et al., 2006). We therefore investigated whether TAM polarization was altered in treated tumors, initially by assessing expression of markers associated with an M2-like, tumor-promoting phenotype (Pyonteck et al., 2013). We found that treatment of gliomas with imipramine but not antiangiogenic agents downregulated an M2-like program, as assessed in bulk tumors and specifically in the total macrophage population in LVRshp53 and PDG models (Figures 4A and 4B, and S5A–S5C). Given that tumor-associated microglia and MDMs each contribute to the immunosuppressive microenvironment in GBM and yet have different transcriptional programs (Klemm et al., 2020), we evaluated the expression of M2-like markers in each cell type. Interestingly, we found interleukin (IL)-10 to be predominantly expressed by MDMs, whereas Arg-1 was significantly higher in microglia (Figure 4C). In addition, we observed major histocompatibility complex (MHC)-II upregulation within the microglial compartment upon IM + anti-VEGF treatment (Figure 4D), possibly facilitating the activation of CD4 T cells.

To functionally assess the inferred reprogramming of TAMs away from an immunosuppressive phenotype, we performed an *ex vivo* assay wherein tumoral CD11b+ cells were co-cultured with activated but antigen-nonspecific T cells, to assess T cell proliferation without the complexity of concomitant antigen-specific killing. The myeloid cells isolated from the untreated and anti-VEGF-treated tumors suppressed CD8 and CD4 T cell proliferation. In contrast, those from IM-treated tumors were significantly less inhibitory (Figure 4E). The combination of anti-VEGF did not alter the effect of IM alone (Figure 4E).

To ascertain if the effects of imipramine were direct, we differentiated bone marrow-derived macrophages (BMDMs) *ex vivo* into M2-like macrophages by subjecting them to polarizing cytokines. IM decreased expression of M2-like signature genes (Figures 4F and S5E), accompanied by increased expression of M1-like markers (Figure 4G). To further investigate the reprogramming capabilities of imipramine, we established a Transwell-based co-culture assay using BMDMs and cancer cells derived from tumors of glioma-bearing mice. Co-cultures of BMDMs with cancer cells induced an M2-like phenotype in macrophages that was diminished upon IM treatment (Figure S5F), concomitant with induction of an M1-like program (Figure S5G).

We further sought to illuminate how imipramine acts mechanistically on macrophages. Our previous publication (Shchors et al., 2015) reported that the combination of IM + TIC promoted autophagic flux via the EPAC branch of the cAMP signaling pathway. However, we excluded cAMP signaling in macrophages as the signaling mechanism herein, in experiments involving EPAC-1/2 inhibitors (Figures S5H and S5I). In recent years, neurotransmitters and their respective receptors have emerged as an important component of the tumor microenvironment that contributes to malignant phenotypes in a variety of cancers (Boilly et al., 2017; Hanoun et al., 2015). Given its role as an antidepressant, we reasoned that imipramine might be affecting neurotransmitter signaling circuits within the myeloid population, thereby promoting pro-inflammatory responses in GBM. We therefore compared the expression of a series of neuronal signals, focusing on receptors for dopamine, serotonin, histamine, and acetylcholine, in the untreated and IM-treated M2-polarized BMDMs. We found the H1 histamine receptor (*Hrh1*) to be significantly upregulated in the M2-like compared with M1-like BMDMs, and markedly downregulated in response to imipramine treatment, reaching the low levels observed in M1-polarized BMDMs (Figure 4H). We further assessed the expression of *Hrh1* in sorted microglia and BMDMs defined as CD49d^-^ and CD49d^+^, respectively (Bowman et al., 2016), and found the histamine receptor to be similarly downregulated by imipramine in both populations of TAMs (Figure 4I). Histamine has been previously shown to induce the upregulation of both mRNA levels and signaling activity of its receptor, and HRH1 antagonists concomitantly suppress both *Hrh1* gene transcription and receptor signaling (Das et al., 2007). To functionally assess this implicated mechanism, we knocked down the expression of *Hrh1* mRNA with siRNAs and found that *siHrh1*-transfected M2-polarized macrophages resembled the expression profile of similarly polarized imipramine-treated BMDMs (Figures 4J and 4K). Conversely, when the M1-polarized macrophages were treated with histamine, we observed a decrease in the expression of M1-like markers (Figure S5J) and an increase in immunosuppressive Arg1 protein levels (Figure S5K). In addition, we evaluated other generic HRH1 antagonists. Both desloratadine and trifluoperazine (TFP) decreased the expression of *Hrh1* in M2-polarized BMDMs to a similar degree as IM, and further reduced the levels of Arg1 and Il10 (Figures S5L and S5M). This pro-inflammatory effect was confirmed *in vivo* with a short-term treatment of GBM-bearing mice with TFP. Isolated myeloid cells were less inhibitory of T cell proliferation compared with untreated controls and presented with a significantly lower expression of immunosuppressive and pro-tumoral markers, combined with a lower expression of *Hrh1* (Figures 4M and S5N), similar to IM-treated tumors. As such, these data reveal the histamine neurotransmitter system to be involved in the suppression of adaptive immunity in GBM.

Finally, we assessed the phagocytic activity of IM-reprogrammed TAMs as a functional component of their immunostimulatory phenotype, given that phagocytic activity has been associated with the capacity to cross-present tumor antigens to CD8^+^ T cells (Tseng et al., 2013, p. 47; von Roemeling et al., 2020). We conducted an *ex vivo* phagocytosis assay with GFP + *Staphylococcus aureus* bioparticles on sorted microglia and MDMs. Treatment with IM contributed to a significant increase in phagocytosis levels in both macrophage subtypes populating tumors in the mesenchymal (LVRshp53) and proneural (PDG) models (Figures 4N and S5O).

Collectively, the data indicate that treatment with imipramine reprograms—via inhibiting the histamine receptor Hrh1—both subtypes of glioma TAMs away from an M2-like immunosuppressive phenotype, thereby functionally contributing to the observed therapeutic benefit of imipramine in combination with VEGF inhibition.

### Associations of antihistamines in the prognosis of human GBM

**Figure 5:**
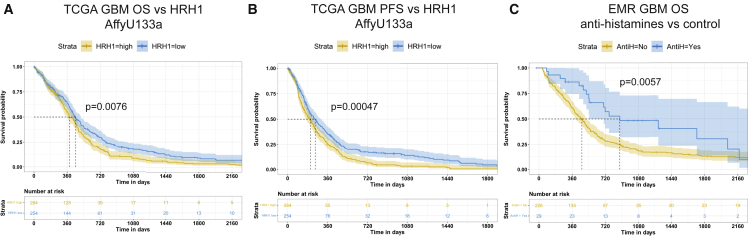
Low HRH1 expression is associated with better survival, and antihistamine treatment is prognostic in GBM patients (A and B) Kaplan-Meier overall survival (A) and progression-free survival (B) analyses of TCGA GBM cohort analyzed with the AffyU133a expression array (n = 539). The blue and yellow shades correspond to the 95% confidence intervals. Patients were split by median expression. p values were calculated based on the Cox proportional hazard model. (C) Kaplan-Meier estimate of the overall survival of GBM patients from a single center who received antihistamine treatment (n = 29, blue curve) or not (n = 226, yellow curve). The shades correspond to the 95% confidence intervals. The analysis was performed with the Mantel-Cox log-rank test.

Considering the implication that HRH1 signaling was programming TAMs in GBM, we evaluated the survival of human GBM patients as a function of differential expression of *HRH1* or of chronic treatment with antihistamines that inhibit HRH1 signaling. First, we queried The Cancer Genome Atlas (TCGA) to determine if the expression of *HRH1* could be associated with differential patient outcomes. Kaplan-Meier survival analysis indicated significantly improved overall survival (Figure 5A) and progression-free survival (Figure 5B) in GBM patients characterized by low tumoral *HRH1* expression. Interestingly, it has recently been reported that patients with melanoma and lung cancer taking antihistamines during ICB treatment exhibited improved responses compared with those who did not (Li et al., 2022). To assess effects of antihistamine use on responses in GBM patients, we analyzed electronic medical records (ERMs) of the University Hospital of Lausanne (CHUV). We found that patients who received antihistamines prior to and during the course of the disease showed a significant reduction in the death rate compared with those who did not (Figure 5C). These data implicate HRH1 signaling in human GBM, consistent with the ascribed mechanism of action of imipramine as an HRH1 inhibitor in mouse GBM models. Interestingly, although a cohort of chronically depressed patients on long-term TCA treatment had a reduced incidence of GBM (Walker et al., 2011), patients with GBM who began taking TCAs post-diagnosis did not show an association with survival, consistent with the lack of appreciable survival benefit of IM monotherapy in overtly tumor-bearing mouse models. It will therefore be of interest to delineate in future studies whether antihistamines can be combined with VEGF pathway inhibitors with or without autophagy-inducing TCAs, and, if so, to consider such combinations for potential clinical evaluation.

### The T cell chemo-attractants CXCL9 and CXCL10 are upregulated in doubly targeted tumors

**Figure 6:**
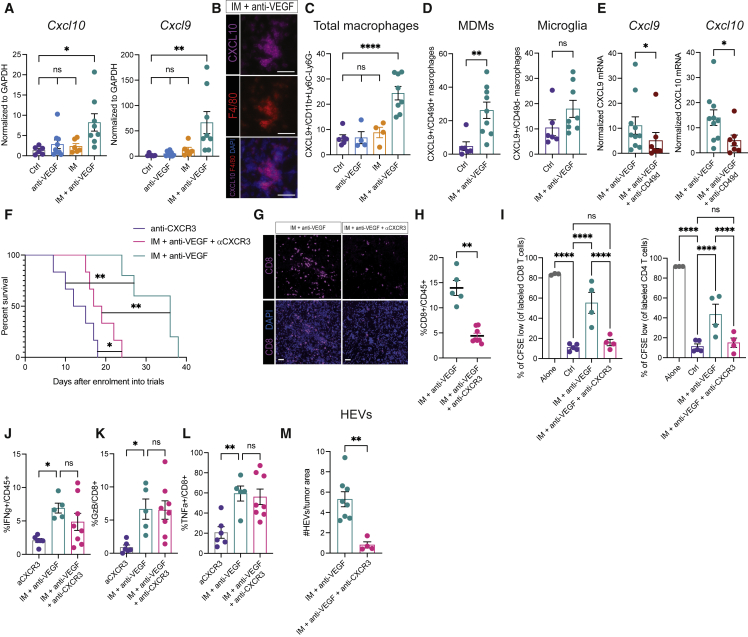
Macrophage-derived CXCR3 ligands are required for the therapeutic benefit conveyed by the combinatorial regimen of IM + anti-VEGF (A) *Cxcl10* and *Cxcl9* expression in bulk tumors. mRNA expression is shown relative to *Gapdh*. Ctrl (n = 6), anti-VEGF (n = 9), IM (n = 6), IM + anti-VEGF (n = 8). (B) Representative image of CXCL10 (magenta), F4/80 (red), and DAPI (blue) staining of LVRshp53 tumors treated with IM + anti-VEGF. Scale bar, 50 μm. Images are illustrative of five to six fields in tissue sections from three different tumors. (C) CXCL9 expression in TAMs in untreated (n = 6) or tumors treated with anti-VEGF (n = 4), IM (n = 4), or IM + anti-VEGF (n = 8) revealed by flow cytometry. (D) CXCL9 expression in MDMs and microglia, evaluated as in (C). (E) mRNA *Cxcl9* and *Cxcl10* expression assessed in bulk tumors treated with IM + anti-VEGF (n = 10) ± αCD49d (n = 7) to selectively deplete MDMs but not microglia. Expression is normalized to *Gapdh* housekeeping gene. (F) Assessing the contribution of CXCR3 function to the survival of LVRshp53 animals subjected to the indicated treatments. Treatment cohorts: αCXCR3 (n = 6), IM + anti-VEGF + αCXCR3 (n = 6), IM + anti-VEGF (n = 5). (G) Representative images of CD8 T cells aimed to assess the effects of αCXCR3. Representative of whole-slide image analysis of three tumors per treatment. Scale bar, 50 mm (H) Flow cytometry analysis of CD8 T cells in tumors subjected to indicated treatments. (I) *Ex vivo* co-culture of tumor-derived CD11b cells and CFSE-labeled CD8 or CD4 T cells. Myeloid cells were isolated from tumors treated with IM + anti-VEGF (n = 4), IM + anti-VEGF + αCXCR3 (n = 4), or untreated Ctrl (n = 5). Each dot represents an average of two or three technical replicates. (J) Minimal effect on IFNγ secretion by CD8 T cells in tumors treated with αCXCR3, IM + anti-VEGF, or the triple combination. (K and L) No effect of αCXCR3 on (K) GzB or (L) TNFα secretion by CD8 T cells co-treated with IM + anti-VEGF. (M) Quantification of immunostaining for HEVs in tumors treated with IM + anti-VEGF (n = 8) or IM + anti-VEGF + αCXCR3 (n = 4). The data are shown as number of HEVs per square millimeter of tumor tissue. (Para break) Data in all quantitative panels are presented as mean ± SEM. ^∗^p < 0.05; ^∗∗^p < 0.01; ^∗∗∗^p < 0.001; ^∗∗∗∗^p < 0.0001; ns, no statistical significance. Statistical analysis by Mann-Whitney test or one-way ANOVA, unless otherwise stated.

Although autophagy has been shown to elicit tumor immunity in some contexts (Ladoire et al., 2016a, 2016b; Michaud et al., 2011; Pietrocola et al., 2016), the mechanisms remain obscure. As an entrée, we profiled untreated versus monotherapy or combo-treated tumors, focusing on cytokines and chemokines. Two chemo-attractants for CD8 T cells—CXCL9 and CXCL10—were substantially upregulated in the IM + anti-VEGF but not the monotherapy arms (Figure 6A). Consistent with a previous report (House et al., 2020), we found via immunostaining and fluorescence-activated cell sorting (FACS) analysis of tumors that CXCL9 and CXCL10 were predominantly expressed by TAMs (Figures 6B and 6C). We therefore assessed the expression of CXCL9 in TAMs derived from recruited MDMs or resident microglia. We found that CXCL9 was significantly upregulated in CD49d+ MDMs by IM + anti-VEGF, whereas no significant induction was observed within the CD49d− microglia (Figure 6D). We confirmed and extended these results in the PDG model, whereby FACS-sorted microglia and MDMs were subjected to mRNA profiling. CD49d+ MDMs were the primary source of CXCL9 and CXCL10 (Figure S6A), significantly upregulated in treated compared with untreated tumors (Figures S6B and S6C). To assess functionality, we applied a CD49d-blocking antibody (Akkari et al., 2020) in IM + anti-VEGF-treated tumors from the LVRshp53 model and observed a significant decrease in the expression of both T cell-recruiting chemokines (Figure 6E).

To investigate the role of CXCL9/CXCL10, we incorporated a blocking αCXCR3 antibody into the above-mentioned therapeutic regimens. Notably, *in vivo* blockade of the CXCR3 receptor significantly reduced the efficacy of IM + anti-VEGF therapy (Figure 6F), and substantially reduced the abundance of tumor-infiltrating CD8 T cells (Figures 6G and 6H). Concordantly, αCXCR3 impaired CD8 and CD4 T cell proliferation in the *ex vivo* co-culture experiments (Figure 6I). However, CXCR3 blockade did not affect the capability of the few remaining T cells to produce cytotoxic cytokines, distinguishing T cell recruitment from activation (Figures 6J–6L). Indeed, tumors treated with imipramine alone had a significant increase in CD8 T cells (Figure 1H) that required VEGF inhibition for activation (see for e.g., Figures 2B–2D). αCXCR3 also obviated the induction of HEVs in combo-treated tumors (Figure 6M), consistent with the role of activated CD8 T cells in their induction (Colbeck et al., 2017; Johansson-Percival et al., 2017).

Taken together, these data indicate that the CXCL9/10-CXCR3 axis is instrumental for the enhanced T cell recruitment into GBM tumors, wherein inhibition of VEGF signaling additionally creates a favorable microenvironment for T cell activation.

### PD-L1 blockade augments CD8 T cell activity and improves the efficacy of IM + anti-VEGF

Despite an impressive response to IM + anti-VEGF therapy, GBM tumors eventually progress and evidently develop resistance (e.g., Figure 1C). Given that the efficacy of the double combination is demonstrably dependent—as shown above—on IFNγ signaling, which is known to stimulate expression of the immune-checkpoint ligand PD-L1 in tumors (Garcia-Diaz et al., 2017), we investigated PD-L1 as a potential factor in adaptive resistance and eventual relapse.

**Figure 7:**
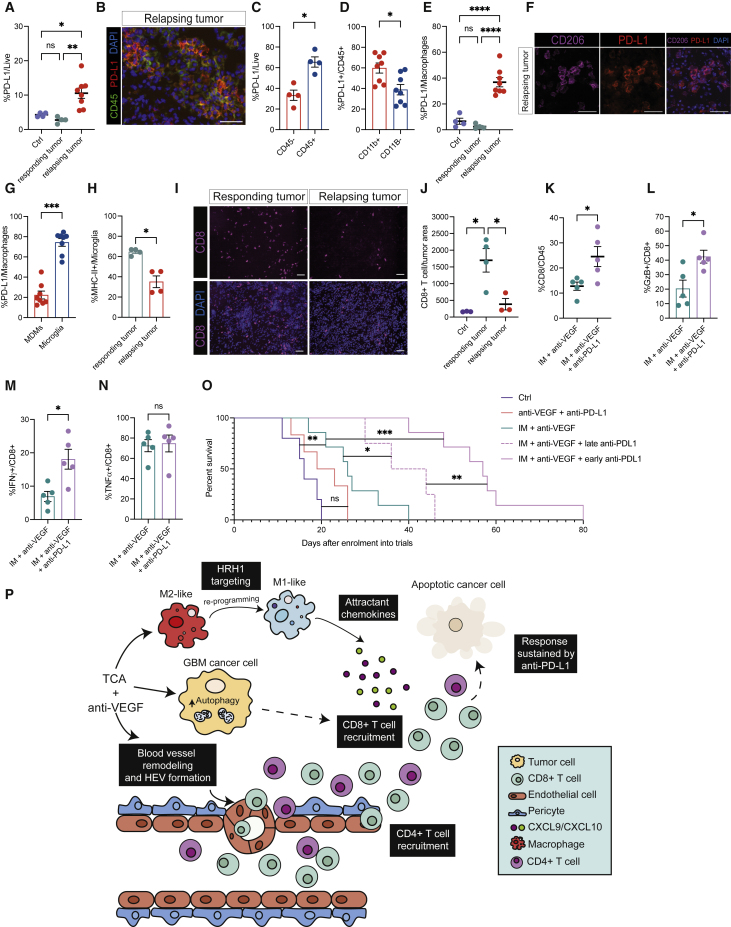
PD-L1 is induced in relapsing tumors and its blockade potentiates T cell function to prolong survival benefit in GBM mice (A) FACS analysis of PD-L1 in the live cell compartment of tumors treated as indicated. Ctrl (n = 4 tumors), responding to IM + anti-VEGF (n = 4), and relapsing from IM + anti-VEGF (n = 8). Responding tumors were collected after 12 days of treatment. Relapsing tumors were collected when mice became symptomatic or when tumors started to re-grow following a stable phase. (B) Representative image of immunostaining to reveal PD-L1 (red), CD45 (green), and DAPI nuclei (blue) in relapsing tumors after IM + anti-VEGF. Scale bar, 50 μm. Assessed in four relapsing tumors, n = 8–10 fields imaged per tumor. (C) Percentage of PD-L1-positive live cells comparing the CD45^−^and CD45^+^ compartments of n = 4 relapsing GBM tumors as revealed by flow cytometry. (D) PD-L1 expression in the CD11b− and CD11b+ compartments of CD45^+^ cells in n = 8 tumors assessed by flow cytometry. (E) Percentage of PD-L1-positive TAMs assessed by FACS. Ctrl (n = 4), responding tumor (n = 4), relapsing tumor (n = 8). (F) Representative immunostaining to reveal PD-L1 expression in TAMs. CD206 (magenta), PD-L1 (red), and DAPI in n = 3 relapsing tumors, 8–10 images per tumor. Scale bar, 50 μm. (G) Expression of PD-L1 in MDMs and microglia of n = 8 relapsing tumors assessed by flow cytometry. (H) MHC-II expression in microglia comparing responding (n = 4) and non-responding tumors (n = 4), assessed by flow cytometry. (I and J) Representative images (I) and quantification (J) of CD8 T cells in untreated (n = 3), responding (n = 4), and relapsing (n = 3) tumors under IM + anti-VEGF treatment. CD8 (magenta) and DAPI-stained nuclei. Scale bar, 50 μm. Each dot indicates the total number of CD8 T cells in an entire tissue section from a tumor. (K) Abundance of CD8 T cells from tumors treated short term with IM + anti-VEGF (n = 5) or IM + anti-VEGF + αPD-L1 (n = 5), assessed by flow cytometry. (L–N) GzB (L), IFNγ (M), and TNFα (N) expression in CD8 T cells from tumors treated as in (K). (O) Assessment of the benefits of early versus late incorporation of anti-PD-L1. Ctrl (n = 5), IM + anti-VEGF (n = 7), IM + anti-VEGF + late anti-PD-L1 (n = 4), IM + anti-VEGF + early anti-PD-L1 (n = 7), anti-VEGF + anti-PD-L1 (n = 6). (P) The combination of a TCA (e.g., imipramine) and VEGF/VEGFR inhibitors induces autophagy in cancer cells and remodels the tumor vasculature, conveying survival benefit for mice bearing GBM. Imipramine reprograms M2-like TAMs to more pro-inflammatory phenotype, via inhibition of histamine receptor signaling. Consequent to the dual treatment, CD8 and CD4 T cells are recruited and activated to evoke their cytotoxic effects. The inclusion of anti-PD-L1 in the therapeutic regimen helps sustain the immune response and increases survival benefit. (Para break) Data in all quantitative panels are presented as mean ± SEM. ^∗^p < 0.05; ^∗∗^p < 0.01; ^∗∗∗^p < 0.001; ^∗∗∗∗^p < 0.0001; ns, no statistical significance. Statistical analysis by Mann-Whitney test or one-way ANOVA, unless otherwise stated.

We observed a substantial increase in PD-L1 when the tumors relapsed in comparison with the otherwise low but detectable levels in control or short-term-treated (responding) tumors (Figure 7A). PD-L1 proved to be upregulated in the immune cell compartment of relapsing tumors (Figures 7B and 7C), principally in TAMs and in particular microglia (Figures 7D–7G). A previous study similarly reported delayed induction of PD-L1 in the TME (Qian et al., 2018). MHC class II expression was upregulated in microglia of tumors responding to IM + anti-VEGF (Figure 4D), and downregulated in non-responding/relapsing tumors (Figure 7H), concomitant with the upregulation of microglial PD-L1 (Figures 7E–7G). Concordantly, the relapsing tumors had reduced infiltration of CD8 T cells and upregulation of the T cell exhaustion markers LAG3 and EOMES (Figures 7I, 7J, S6D, and S6E), of which LAG3 is also considered a functionally important immune checkpoint in cancer (Aroldi et al., 2022, p. 3).

These results motivated evaluation of therapeutic combinations with an anti-PD-L1 antibody. We first characterized GBM tumors after short-term treatment wherein anti-PD-L1 was incorporated into the therapeutic regimen on the same day as IM + anti-VEGF, when PD-L1 is expressed at low levels (Figure 7A). Concomitant immune-checkpoint blockade significantly increased CD8 T cell abundance (Figure 7K). IFNγ and GzB were significantly increased, whereas TNFα was unchanged (Figures 7L–7N). Next, we enrolled animals into longer trials where anti-PD-L1 was incorporated concurrently or subsequently into the IM + anti-VEGF therapeutic regimen. We observed a significant improvement in overall survival in both cases (Figure 7O). However, the most pronounced benefit was observed when PD-L1 blockade was initiated on the same day as IM + anti-VEGF (Figure 7O). These data are congruent with previous studies in different pre-clinical models suggesting that early/initial inclusion of immune-checkpoint inhibitors is more effective than incorporation later in the course of therapeutic treatment (Cloughesy et al., 2019; Liu et al., 2016). Interestingly, the combination of anti-VEGF + anti-PD-L1 (without IM) did not improve the overall survival of GBM-bearing mice (Figure 7O), consistent with the failed clinical trials combining PD-1/PD-L1 checkpoint inhibitors with antiangiogenic agents (NCT02337491, NCT03291314), highlighting the importance of imipramine for the observed efficacy of this novel triple therapy (Figure 7O). We also assessed the therapeutic benefit of combining IM with anti-PD-L1 but without anti-VEGF. Interestingly, this combination had a similar survival advantage to IM + anti-VEGF (Figure S6F), potentially congruent, given the inhibitory effect of IM on HRH1 signaling described above, with a previous report describing better outcomes of human melanoma and lung cancers patients on ICB therapies who were concurrently receiving antihistamines (Li et al., 2022). The underlaying mechanisms of response to the IM + anti-PD-L1 therapy warrants further investigation in our GBM models. The substantial survival benefit of the IM + anti-VEGF + anti-PD-L1 triple therapy is nevertheless still limited in duration, implicating other mechanisms of eventual adaptive resistance to this therapeutic regimen, a topic that stands as an important question (and opportunity) for the future (van Elsas et al., 2020).

In conclusion, TCAs, exemplified by imipramine, have been revealed herein to elevate levels of ostensibly immunostimulatory autophagy in cancer cells and to reprogram immunosuppressive macrophages in the TME to express chemokines that attract otherwise rare CD8 T cells, which are expanded and activated by the inclusion of VEGF pathway inhibitors and further sustained by immune-checkpoint blockade, collectively contributing to the significant therapeutic efficacy of this innovative mechanism-guided therapeutic strategy (Figure 7P).

## Discussion

This investigation has revealed a mechanistically intriguing therapeutic co-targeting strategy for GBM brain cancer, in which autophagy is elevated in cancer cells concomitant with reprogramming tumor-promoting macrophages and modifying the angiogenic vasculature. Consistent with clinical experience (Chinot et al., 2014; Duerinck et al., 2018; Gilbert et al., 2014; Hutterer et al., 2014), the anti-VEGF antibody B20S (a mouse analog of the clinically approved antibody drug bevacizumab) as well as tyrosine kinase inhibitors of the VEGF receptors (axitinib and sunitinib) had no therapeutic efficacy, despite the on-target effect of modestly reducing the density of the tumor vasculature. In contrast, when combined with the TCA imipramine, VEGF/VEGFR inhibitors contributed to significant therapeutic benefit in pre-clinical trials involving multiple *de novo* mouse models of GBM. Unexpectedly, the combination unleashed a potent anti-tumoral immune response involving inflammation by CD8 and CD4 T cells that proved to be the driving force in therapeutic efficacy. The combination of imipramine and a VEGF/VEGFR inhibitor was crucial for the accumulation and activation of CD8 and CD4 T cells, which were otherwise rare and suppressed. Importantly, inclusion of depleting antibodies for both CD8 and CD4 T cell populations completely abrogated therapeutic benefit. As such, the combination of IM + VEGF/VEGFR pathway inhibition is immunostimulatory, a new therapeutic modality for a lethal tumor type heretofore refractory to immune intervention.

Our investigation has revealed new biological activities of TCAs. First, imipramine as monotherapy elicits increased infiltration of CD8 and CD4 T cells into gliomas, recruitment that is dependent on the elevated levels of autophagy, as confirmed by a knockdown of the autophagic regulatory gene ATG3 in cancer cells, consistent with previous reports that autophagy can be immunostimulatory (Ramakrishnan et al., 2012; Rao et al., 2014). As such, further studies to delineate the molecular mechanisms of immunostimulatory autophagy are warranted. Second, imipramine reprogramed TAMs in the glioma tumor microenvironment, converting immunosuppressive microglia and MDMs into pro-inflammatory macrophages, each of which participates distinctly in the recruitment of T cells, in part by expressing the pro-inflammatory chemokines CXCL9 and CXCL10, as validated by inhibition of their receptor CXCR3. This reprogramming of macrophages demonstrably involves the inhibition by IM of histamine H1 receptor expression in immunosuppressive TAMs; inhibition of Hrh1 is on target, consistent with imipramine’s mechanism of action as an antidepressant in the brain. Provocatively, the use of antihistamines is seemingly beneficial for GBM patients, given that chronic ingestion was associated with a better outcome. Our study has not addressed the possibility that imipramine (and antihistamines) may also modulate the tumor-promoting activity of neutrophils and their granulocytic myeloid progenitors, which have been implicated in a mouse model of GBM (Magod et al., 2021).

While imipramine was pro-inflammatory as monotherapy, the degree of immune cell infiltration was modest, and the CD8 T cells were largely inactive. In marked contrast, when a VEGF pathway inhibitor was included, the infiltration of CD8 and CD4 T cells was increased, and the CD8 T cells were activated; in addition, cytotoxic CD4 T cells were detected, implicating them both in supporting CD8 T cell function and in directly killing glioma cancer cells. The VEGF pathway inhibitors had several effects salient to their combinatorial benefit. First, the levels of autophagy were increased over that produced by IM alone. Second, the aberrant tumor vasculature was remodeled into more normal-like morphology, with induction of HEVs, features known to facilitate T cell infiltration into tumors (Allen et al., 2017; Lanitis et al., 2015; Schmittnaegel et al., 2017). Notably, VEGF inhibitors are showing benefits in clinical trials in combination with immunomodulatory agents (Hodi et al., 2014) (NCT02210117, NCT02348008, NCT01633970), albeit not in GBM. Consistently, there is no benefit if imipramine is excluded in our GBM models, thus mirroring clinical experiences involving VEGF pathway inhibitors and immune-checkpoint inhibitors, either alone or in combination (NCT02337491, NCT03291314). Finally, the reduction in vascular density (inhibition of angiogenesis and vascular pruning) produced regions of hypoxia, wherein a subset of CD8 cells were preferentially localized, consistent with reports that hypoxia can contribute to T cell activation (de Almeida et al., 2020; Doedens et al., 2013; Xu et al., 2016).

The therapeutic benefit produced by the combination was notable, but nevertheless limited, implicating adaptive resistance. The immune-checkpoint ligand PD-L1 was upregulated in relapsing tumors, consistent with a role in CD8 T cell exhaustion and therapeutic resistance (Jiang et al., 2015). We therefore incorporated a PD-L1 blocking antibody into the therapeutic regimen, which markedly enhanced survival. Despite almost doubling the survival benefit, the triple therapy eventually failed, implicating yet other resistance mechanisms worthy of future investigation. Other future investigations that might incentivize clinical trials include the development of standard of care (SoC) models using TMZ and RT that are tractable for testing the double and triple combos in a quasi-second-line setting (without surgery), as well as first line by including them up front with SoC agents.

Finally, although we suspect that the hyperactivation of autophagy may be relatively specific to GBM, there is interesting promise that the combination of IM + anti-VEGF (± immune-checkpoint blockade) could have broader applicability, as illustrated by our finding that imipramine also promotes CD8 T cell influx in a mouse model of BRAF-induced melanoma. These results motivate future studies to assess the therapeutic utility of imipramine in melanoma and other tumor types as an enhancer of tumor immunity in combination with VEGF/VEGFR inhibitors and various immunotherapeutic modalities.

In conclusion, the combination of three classes of clinically approved drugs resulted in remarkable therapeutic benefit in a mouse model of GBM, by virtue of concordantly modifying multiple features of the otherwise immunosuppressive glioma tumor microenvironment, thereby rendering it susceptible to efficacious immune attack (Figure 7P). Given the dismal prognosis for GBM patients, these conceptual findings motivate consideration of clinical trials aimed to evaluate TCAs such as imipramine combined with VEGF/VEGFR pathway inhibitors and immune-checkpoint blockade. Toward that end, our results have motivated a proof-of-concept clinical trial in second-line GBM patients (https://themarkfoundation.org/portfolio/a-proof-of-concept-clinical-trial-of-an-innovative-new-therapy-for-glioblastoma/), which will begin to assess the translational potential of this intriguing new therapeutic strategy.

## STAR★Methods

### Key resources table

**Table:** 

REAGENT or RESOURCE	SOURCE	IDENTIFIER
**Antibodies**		

*InVivo*MAb anti-mouse CD8α (clone 2.43)	BioXCell	Cat #: BE0061; RRID: AB_1125541
*InVivo*MAb anti-mouse CD4 (clone GK1.5)	BioXCell	Cat #: BE0003-1; RRID: AB_1107636
*InVivo*Plus anti-mouse IFNγ (clone XMG1.2)	BioXCell	Cat #: BP0055; RRID: AB_1107694
*InVivo*MAb anti-mouse VEGFR-2 (clone DC101)	BioXCell	Cat #: BE0060; RRID: AB_1107766
*InVivo*MAb anti-mouse CXCR3 (CD183) (clone CXCR3-173)	BioXCell	Cat #: BE0249; RRID: AB_2687730
*InVivo*MAb anti-mouse/human VLA-4 (CD49d) (clone PS/2)	BioXCell	Cat #: BE0071; RRID: AB_1107657
*InVivo*MAb anti-mouse PD-L1 (B7-H1)	BioXCell	Cat #: BE0101; RRID: AB_10949073
FITC TNF alpha Rat anti-mouse (cloneMP6-XT22)	eBioscience	Cat #: 11-7321-82; RRID: AB_465418
PE Granzyme B anti-mouse (clone NGZB)	eBioscience	Cat #: 12-8898-82; RRID: AB_10870787
PerCP-Cyanine5.5 CD3e anti-mouse (clone 145-2C11)	eBioscience	Cat #: 45-00310-82; RRID: AB_1107000
PE/Cyanine7 CD8a anti-mouse (clone 53-6.7)	Biolegend	Cat #: 100722; RRID: AB_312760
Biotin CD11b anti-mouse (clone M1/70)	eBioscience	Cat#: 13-0112-82; RRID: AB_466359
APC IFN-γ anti-mouse (clone XMG1.2)	Biolegend	Cat #: 505810; RRID: AB_315403
APC/Cyanine7 CD45 anti-mouse (clone 30-F11)	Biolegend	Cat#: 103116; RRID: AB_312980
FITC CD62L anti-mouse (clone MEL-14)	Biolegend	Cat#: 104406; RRID: AB_313093
PerCP-Cyanine5.5 CD11b anti-mouse (clone M1/70)	eBioscience	Cat#: 45-0112-82; RRID: AB_953558
Pacific Orange™ CD8a anti-mouse (clone 5H10)	Invitrogen	Cat#: MCD0830, RRID: AB_10376311
APC CD45 anti-mouse (clone 30-F11)	BD Biosciences	Cat#: 559864; RRID: AB_398672
Pe-Cyanine7 CD3e (clone 145-2C11)	eBioscience	Cat#: 25-0031-81; RRID: AB_469572
Alexa Fluor® 488 Anti-Stat5 (pY694) anti-mouse (clone 47/Stat5(pY694))	BD Biosciences	Cat#: 612598; RRID: AB_399881
PE TCF-7/TCF-1 anti-mouse (clone S33-966)	BD Biosciences	Cat#: 564217; RRID: AB_2687845
Pacific Blue™ Ki-67 anti-mouse	Biolegend	Cat#: 652422; RRID: AB_2564490
PerCP-Cyanine5.5 Foxp3 anti-mouse (clone FJK-16s)	eBioscience	Cat#: 45-5773-82; RRID: AB_914351
APC CD3e anti-mouse (clone 145-2C11)	Biolegend	Cat#: 100312; RRID: AB_312677
Biotin CD4 anti-mouse (clone GK1.5)	Biolegend	Cat#: 100404; RRID: AB_312689
Pe-Cyanine7 CD4 anti-mouse (clone RM4-5)	Biolegend	Cat#: 100528; RRID: AB_312729
Brilliant Violet 510™ CD11b anti-mouse/human (clone M1/70)	Biolegend	Cat#: 101245; RRID: AB_2561390
Alexa Fluor® 488 CRACC/SLAMF7 anti-mouse	R&D Systems	Cat#: FAB46281G
Brilliant Violet 421™ LAP (TGF-β1) anti-mouse (clone TW7-16B4)	Biolegend	Cat#: 141407; RRID: AB_2561580
APC-eFluor™ 780 CD45 anti-mouse (clone 30-F11)	Biolegend	Cat#: 47-0451-82; RRID: AB_1548781
Brilliant Violet 650™ CD223 (LAG-3) anti-mouse (clone C9B7W)	Biolegend	Cat#: 125227; RRID: AB_2687209
Brilliant Violet 421™ Eomes anti-mouse (clone X4-83)	BD Biosciences	Cat#: 567166; RRID: AB_2916483
APC HIF-1 alpha anti-mouse/anti-human (clone 241812)	R&D Systems	Cat#: IC1935A; RRID: AB_ 1061580
Alexa Fluor™ 488 Arginase 1 anti-mouse/anti-human (clone A1exF5)	eBioscience	Cat#: 53-3697-82; RRID: AB_2734831
APC/Cyanine7 IL-10 anti-mouse (clone JES5-16E3)	Biolegend	Cat#: 505036; RRID: AB_2566331
PerCP-Cyanine5.5 Ly-6C anti-mouse (clone HK1.4)	eBioscience	Cat#: 45-5932-82; RRID: AB_2723343
Pacific Blue™ Ly-6G anti-mouse (clone 1A8)	Biolegend	Cat#: 127612; RRID: AB_2251161
Alexa Fluor® 647 CD49d anti-mouse (clone R1-2)	Biolegend	Cat#: 103614; RRID: AB_528837
FITC CD49d anti-mouse (clone R1-2)	Biolegend	Cat#: 103605; RRID: AB_313037
Alexa Fluor® 647 CXCL9 anti-mouse (clone MIG-2F5.5)	Biolegend	Cat#: 515606; RRID: AB_1877135
FITC I-A/I-E anti-mouse (clone M5/114.15.2)	Biolegend	Cat#: 107606; RRID: AB_313321
PE CD274 (B7-H1, PD-L1) anti-mouse (clone 10F.9G2)	Biolegend	Cat#: 124308; RRID: AB_2073556
PE-Cyanine7 CD11b anti-mouse (clone M1/70)	eBioscience	Cat#: 25-0112-82; RRID: AB_469588
PerCP/Cyanine5.5 CD86 anti-mouse (clone GL-1)	Biolegend	Cat#: 105028; RRID: AB_2074994
APC IL-6 anti-mouse (clone MP-5-20F3)	Biolegend	Cat#: 504508; RRID: AB_10694868
PE IL-1α anti-mouse (clone ALF-161)	Biolegend	Cat#: 503203; RRID: AB_315281
FITC CD8a anti-mouse (clone 53-6.7)	Biolegend	Cat#: 100706; RRID: AB_312745
Alexa Fluor® 647 CD8a anti-mouse (clone 53-6.7)	Biolegend	Cat#: 100724; RRID: AB_389326
Alexa Fluor® 647 CD4 anti-mouse (clone RM4-5)	Biolegend	Cat#: 100530; RRID: AB_389325
FITC CD31 anti-mouse (clone MEC 13.3)	BD Biosciences	Cat#: 553372; RRID: AB_394818
PE CD31 anti-mouse (clone MEC 13.3)	BD Biosciences	Cat#: 553373; RRID: AB_394819
Alexa Fluor® 647 CD206 anti-mouse (clone C068C2)	Biolegend	Cat#: 141712; RRID: AB_10900420
PE F4/80 anti-mouse (clone BM8)	eBioscience	Cat#: 12-4801-82; RRID: AB_465923
FITC Ki67 anti-mouse (clone 16A8)	Biolegend	Cat#: 652409; RRID: AB_2562140
MECA-79	Santa Cruz Biotechnology	Cat#: sc-19602; RRID: AB_627143
CXCL10/IP-10/CRG-2 anti-mouse	R&D Systems	Cat#: AF-466-NA; RRID: AB_2292487
Cleaved Caspase-3 (Asp175) (clone 5A1E)	Cell Signaling Technology	Cat#: 9664; RRID: AB_2070042
CD140b (PDGFRB) anti-mouse (clone APB5)	eBioscience	Cat#: 14-1402-82; RRID: AB_467493
LC3 anti-mouse (clone 5F10)	Nanotools	Cat#: 0231-100; RRID: AB_2722733
Desmin	Abcam	Cat#: ab15200; RRID: AB_301744
LAMP1	Abcam	Cat#: ab24170; RRID: AB_775978
Arginase I (N-20)	Santa Cruz Biotechnology	Cat#: sc-18351; RRID: AB_2258542
MMR/CD206 anti-mouse	R&D Systems	Cat#: AF2535; RRID: AB_2063012
IL-10 anti-mouse (clone JES052A5)	R&D Systems	Cat#: MAB417; RRID: AB_2125085
ATG3	Cell Signaling Technology	Cat#: 3415; RRID: AB_2059244
HSP-90 (F-8)	Santa Cruz Biotechnology	Cat#: sc-13119; RRID: AB_675659
GAPDH (clone 14C10)	Cell Signaling Technology	Cat#: 2118; RRID: AB_561053
Polyclonal Rabbit Anti-Goat Immunoglobulins/HRP antibody	DAKO	Cat#: P0449; RRID: AB_2617143
Goat Anti-Rabbit Immunoglobulins/HRP antibody	DAKO	Cat#: P0448; RRID: AB_2617138
Goat Anti-Mouse Immunoglobulins/HRP antibody	DAKO	Cat#: P0447; RRID: AB_2617137
Anti-rat IgG, HRP-linked antibody	Cell Signaling Technology	Cat#: 7077; RRID: AB_10694715
Alexa Fluor™ 546 goat anti-Rabbit IgG (H+L) Highly Cross-Adsorbed Secondary Antibody	Invitrogen	Cat#: A-11035; RRID: AB_2534093
Alexa Fluor™ 647 donkey anti-Goat IgG (H+L) Cross-Adsorbed Secondary Antibody	Invitrogen	Cat#: A-21447; RRID: AB_2535864
Alexa Fluor® 647 donkey Anti-Rat IgG H&L	Abcam	Cat#: ab150155; RRID: AB_2813835
Purified anti-mouse CD16/32	Biolegend	Cat#: 101302; RRID: AB_312801
Lycopersicon Esculentum (Tomato) Lectin (LEL, TL), DyLight 594	Invitrogen	Cat#: L32471
Lectin Kit I, Biotinylated	Vector Laboratories	Cat#: BK-1000; RRID: AB_2336252

**Bacterial and virus strains**		

One Shot™ TOP10 Chemically Competent *E. coli*	Invitrogen	Cat#: C404010
LVRshp53 second generation lentivirus	In house	This paper

**Chemicals, peptides, and recombinant proteins**		

Imipramine hydrochloride	Sigma-Aldrich	Cat#: I0899
(S)-(+)-Clopidogrel hydrogensulfate	Sigma-Aldrich	Cat#: SML0004
Axitinib (AG 013736)	Selleckchem	Cat#: S1005
Sunitinib (SU11248) malate	Selleckchem	Cat#: S1042
Recombinant B20S (anti-VEGF)	In house	This paper
Desloratadine	Sigma-Aldrich	Cat#: D1069
Trifluoperazine hydrochloride	Sigma-Aldrich	Cat#: T6062
HJC0197	Cayman Chemicals	Cat#: CAY-19092
CE3F4	Cayman Chemicals	Cat#: CAY-17767
Histamine	MedChemExpress	Cat#: Y-B1204
CFSE Cell Division Tracker Kit	Biolegend	Cat#: 422701
Dynabeads™ Mouse T-Activator CD3/CD28 for T-Cell Expansion and Activation	Gibco	Cat#: 11452D
Brilliant Violet 421™ Streptavidin	Biolegend	Cat#: 405226
Streptavidin, Pacific Orange™ conjugate	Invitrogen	Cat#: S32365
DAPI	Roche	Cat#: 10236276001
LIVE/DEAD™ Fixable Violet Dead Cell Stain Kit, for 405 nm excitation	Invitrogen	Cat#: L34964
LIVE/DEAD™ Fixable Red Dead Cell Stain Kit, for 488 nm excitation	Invitrogen	Cat#: L23102
PrimeScript RT Master Mix	Takara	Cat#: RR036A
miRNeasy Micro Kit	Qiagen	Cat#: 217084
miRNeasy Mini Kit	Qiagen	Cat#: 217004
QuantiNova SYBR Green PCR Kit	Qiagen	Cat#: 208052
Pierce™ BCA Protein Assay Kit	Thermo Scientific™	Cat#: 23225
Fluorescence Mounting Medium	DAKO	Cat#: S302380
Recombinant Mouse M-CSF (carrier-free)	Biolegend	Cat#: 576406
ACK Lysing Buffer	Gibco	Cat#: A1049201
Dispase® II (neutral protease, grade II)	Roche	Cat#: 04942078001
DNase I recombinant, RNase-free	Roche	Cat#: 04716728001
Collagenase A	Roche	Cat#: 10103578001

**Critical commercial assays**		

cAMP Assay Kit (Competitive ELISA)	Abcam	Cat#: ab65355
EasySep™ Mouse CD11b Positive Selection Kit II	StemCell Technologies	Cat#: 18970
EasySep™ Mouse CD4^+^ T Cell Isolation Kit	StemCell Technologies	Cat#: 19852
EasySep™ Mouse CD8^+^ T Cell Isolation Kit	StemCell Technologies	Cat#: 19853
CD31 MicroBeads, mouse	Miltenyi Biotec	Cat#: 130-097-418
Myelin Removal Beads II, human, mouse, rat	Miltenyi Biotec	Cat#: 130-096-731
pHrodo™ Green *S. aureus* Bioparticles™ Conjugate for Phagocytosis	Invitrogen	Cat#: P35367

**Deposited data**		

TCGA glioblastoma (with AffyU133a array)	Goldman et al., 2020	https://xena.ucsc.edu
CHUV EMRs	This paper	raw data available upon request

**Experimental models: Cell lines**		

293T/17 [HEK 293T/17]	ATCC	Cat#: CRL-11268™; RRID: CVCL_1926
Mouse GBM-derived cancer cells	Derived in house from the LVRshp53 model	This paper
DF-1:RCAS-hPDGF-B-HA	provided by J.A. Joyce	

**Experimental models: Organisms/strains**		

Mouse: FVB.GFAP-Cre	In house	This paper
Mouse: GFAP-HRasV12; GFAP-CRE; GFAP-LUC; p53^flox/wt^	In house	This paper
Mouse: GFAP-HRasV12; GFAP-CRE; GFAP-LUC; p53^flox/flox^	In house	This paper
Mouse: C57BL/6J (JAX® Mice Strain)	Charles River	Strain Code: 632
Mouse: FVB/NCrl	Charles River	Strain Code: 207
Mouse: Fox Chase SCID Beige Mouse	Charles River	Strain code: 250
Mouse: iBIP2 (*TetO-BRAF*^*v600e*^*, Tyr-CreERT2, Rosa26-Lox-Stop-Lox-rtTA, Pten-fl/fl, Cdkn2a-fl/fl)*	In house	This paper
Mouse: Nestin-Tv-a;*Ink4a/Arf*^−/−^ (Tg(NES-TVA)J12Ech; Cdkn2a^tm1Rdp^)	provided by J.A. Joyce	

**Oligonucleotides**		

MISSION® siRNA Universal Negative Control #1	Sigma-Aldrich	Cat#: SIC001
MISSION® Predesigned si*Hrh1*	Sigma-Aldrich	NM_008285; siRNA ID: SASI_Mm01_00058730
MISSION® Predesigned si*Hrh1*	Sigma-Aldrich	NM_008285; siRNA ID: SASI_Mm01_00058731
Primers: see Table S1		

**Recombinant DNA**		

ATG3 Mission shRNA plasmid	Sigma-Aldrich	ID: TRCN0000247440
pMD2G	Addgene	Cat#: 12259
pCMVR8.74	Addgene	Cat#: 22036
pTomo H-rasV12-shp53-Luc	provided by I. Verma	

**Software and algorithms**		

QuPath (version 0.2.3)	Bankhead et al., 2017	https://qupath.github.io
FlowJo (version 10.7.2)	BD	https://www.flowjo.com
RStudio (version 2022.02.1 Build 461)	The R Foundation	https://www.r-project.org/
survminer (version 0.4.9)		https://cran.r-project.org/web/packages/survminer/index.html
GraphPad Prism (version 9.3.1)	Dotmatics	https://www.graphpad.com

### Resource availability

#### Lead contact

Further information and requests for resources should be directed to the lead contact, Douglas Hanahan (douglas.hanahan@epfl.ch).

#### Materials availability

This study did not generate new unique reagents.

### Method details

#### Study design

This study was designed to assess the potential antitumoral impact of combined therapy with autophagy-hyperactivating agents and angiogenesis inhibitors in glioblastoma. We evaluated the efficacy of combinatorial treatments principally in a lentivirally-induced (LVRshp53) model, as well as in transgenic (GRLp53het & GRLp53flko (Shchors et al., 2015), PDG (Hambardzumyan et al., 2009; Pyonteck et al., 2013)) and orthotopic cell transplant (GL261 (Szatmári et al., 2006)) mouse models of glioblastoma and focused on their potential role in tumor vasculature normalization and immunomodulatory actions. All animal studies were performed in accordance with protocols approved by the Veterinary Authorities of the Canton Vaud.

The design of the experimental trials and follow-up analyses is presented in the Materials and Methods. For detailed information on sample size and statistical methods, please see the presented figures or associated figure legends.

#### Mouse models

To initiate gliomas in LVRshp53 mice, GFAP-Cre mice in the FVBn genetic background (both males and females) were intracranially injected at 8–11 weeks of age with the pTomo HRasV12-Luc-shp53 lentivirus, using a stereotactic frame under full anesthesia with a mix of Fentanyl, Midazolam and Medetomidine. The injections were performed using the following coordinates: 2.0 mm anterior/posterior, 1.5 mm medial/lateral, and 2.3 mm dorsal/ventral from the bregma. A small volume of virus was injected (0.8 μL, 1 × 10^8^ international units) at a rate of 0.1 μL/min with an automatic pump. Animals were revived from anesthesia with a triple-shot mix of Naloxon, Flumazenil and Atipamezol. The generation and characterization of the GRLp53het (GFAP-HRasV12; GFAP-CRE; GFAP-LUC; p53^flox/wt^) and GRLp53flko (GFAP-HRasV12; GFAP-CRE; GFAP-LUC; p53^flox/flox^) mouse lines have been described previously (Shchors et al., 2015), as has the initiation of PDG glioma tumors (Pyonteck et al., 2013). For the orthotopic transplantation model involving LVRshp53 tumor-derived glioma cells, 50,000 cells in Neurobasal medium, (ThermoFisher Scientific) were engrafted similarly to the lentivirus into anaesthetized FVBn or SCID animals. For the GL261 syngeneic model, C57BL/6 were intracranially transplanted with 100,000 cells using the same protocol. A transgenic mouse model (iBIP2) of mutant BRAF-driven melanoma was generated from the previously described iBIP model (Neubert et al., 2018) by replacing the germ-line knockout of *Cdkn2A* with a floxed allele, producing a mouse of the following genotype: *TetO-BRAF*^*v600e*^*, Tyr-CreERT2, Rosa26-Lox-Stop-Lox-rtTA, Pten-fl/fl, Cdkn2a-fl/fl*. Topical application of tamoxifen induces the development of melanoma, as will be described in further depth elsewhere.

#### Statistics

Statistical analyses were carried out using GraphPad Prism 9. Data are reported as mean ± SEM, unless otherwise stated in figure legends. p-values are reported in the figures or figure legends and were assessed with unpaired Mann-Whitney test to compare the means of two groups or one-way ANOVA for multiple groups comparisons, unless otherwise stated. For survival analyses, log-rank (Mantel–Cox) test was performed. Statistical significance is indicated as ^∗^p<0.05, ^∗∗^p<0.01, ^∗∗∗^p<0.001, ^∗∗∗∗^p<0.0001.

#### Production and titration of lentiviral particles

High-titer lentiviral particles were produced as previously described (Salmon and Trono, 2006). Briefly, 293T cells were seeded a day before the transfection at 9 × 10^6^/15-cm dish. The transfection mix for 1 plate was obtained by mixing 22.5 μg transfer vector plasmid (provided by I. Verma, pTomo H-rasV12-shp53-Luc), 7.9 μg pMD2G (Addgene, 12259) and 14.6 μg pCMVR8.74 (Addgene, 22036) with 0.66 mL 0.1XTE, 0.35 mL H_2_O, 113 μL CaCl_2_ 2.5M and 1.14 mL 2X HeBS. The precipitate was added dropwise and the dishes incubated overnight. On the next day, the media was replaced and the virus was collected 3–4 times in 8–12 hour increments. The supernatant was pooled, filtered using a 0.22 μm filter unit and ultracentrifuged at 22,000 rpm (Beckman Coulter, SW32Ti rotor). The viral particles were then resuspended in PBS and stored at −80°C. For the titration of the particles, HEK 293 T cells were transduced with various amounts of virus and analyzed by FACS based on mCherry reporter expression (Salmon and Trono, 2006).

#### Cell culture

For intracranial injections and co-culture experiments, mouse brain cancer cells were harvested from LVRshp53 animals and cultured as described previously (Shchors et al., 2015). Melanoma cancer cells were derived from iBIP2 transgenic mouse model and cultured in RPMI medium supplemented with 10% fetal bovine serum, 1% penicillin-streptomycin and 2 μg/mL doxycycline. To generate the knockdown of ATG3 in mouse GBM cells, we used a predesigned shRNA (TRC clone ID: TRCN0000247440, MISSION shRNA, Sigma-Aldrich). The viral particles were produced as above and added to the cells for overnight incubation with polybrene at 8 μg/mL. The next day, the media was changed and the cells allowed to recover for 48 hours before selection with puromycin at 10 μg/mL.

#### Bioluminescent monitoring

Tumor growth was monitored with bioluminescence, starting at week 8 for the GRLp53het and GRLp53flko mice and at weeks 2 post-surgery for the LVRshp53 lentivirus model. Images were obtained five minutes after injection of a PBS solution containing firefly D-luciferin potassium salt (Biosynth), using an IVIS-100 Imaging System, applying the following parameters: medium binning, open emission filter, F/stop 1, and 1-minute exposure. Luminescent images were analyzed using Living Image 3.2 Analysis software. The criteria for enrollment into therapeutic trials was a luminescent value of 3.5 to 4.5×10^6^ photons per second per square centimeter for GRLp53het and GRLp53flko mice and 1 to 1.5×10^6^ photons per second per square centimeter for LVRshp53 mice in the brain regions of interest.

#### MRI imagining

Tumor growth of PDG and GL261 gliomas was monitored with T2-weighted 1H MRI scans on a 3T MRI machine (Bruker). The mice were enrolled into therapeutic trials when the tumors reached approximately 15mm^3^ of volume.

#### Therapeutic trials

All of the animals enrolled in the trials were included in the analyses. Mice were randomly assigned to the experimental cohorts. Imipramine (Sigma I0899) was prepared in 0.9% NaCl physiological solution and administered orally once a day at 40 mg/kg. Ticlopidine (Sigma T6654) and clopidogrel (Sigma SML0004) solutions were formulated in 0.9% NaCl and injected intraperitoneally once a day at 1 mg/kg. The anti-mouse VEGF monoclonal antibody (B20S), a mouse biosimilar to anti-human VEGF (bevacizumab), was generated based on a previously published protocol using the B20-4.1 sequence (Liang et al., 2006). The piggy-Bac transposon system was utilized to produce recombinant B20S in 293 cells (32). B20S was affinity-purified, and stored in a buffer comprising of 50 mM sodium phosphate and 150 mM NaCl (pH 7.0). Mice were dosed twice a week at 20 mg/kg. Axitinib (Selleckchem S1005) was prepared in 0.5% carboxymethylcellulose/H_2_O-HCl (pH 2.0) and administered by oral gavage twice daily at 30 mg/kg. Sunitinib (Selleckchem S1042) was formulated in 0.5% carboxymethylcellulose, 0.4% Tween 80, 1.8% NaCl, 0.9% benzyl alcohol dissolved in reverse osmosis deionized water (pH 6.0) and administered once daily by oral gavage at 40 mg/kg. Trifluoperazine hydrochloride (Sigma T6062) in saline was administered intraperitoneally once a day at 40 mg/kg. The anti-mouse CD8 (clone 53-6.72), the anti-mouse CD4 (clone GK1.5), the anti-mouse IFNγ (clone XMG1.2), the anti-mouse VEGFR2 (clone DC101), the anti-mouse CXCR3 (clone CXCR3-173), the anti-mouse VLA-4 (CD49d, clone PS/2), and the anti-PD-L1 (clone 10F.9G2) monoclonal antibodies were purchased from BioXcell and intraperitoneally injected twice a week at 250 μg/mouse/dose.

#### Harvesting of mouse tissues

Animals were euthanized at specific time points described in the figure legends. For immunofluorescence staining, mice were perfused by intracardiac inoculation with phosphate-buffered saline (PBS, 10mM, pH 7.4). Brain tissues were embedded in O.C.T (Tissue-Tek) and sectioned with a cryostat (CM1950 or CM3050S Leica) to produce 8μm or 16μm-thick tissue sections. For immunohistochemical analyses, each mouse was treated before euthanasia by trans-cardiac perfusion with PBS and formalin. Excised whole brains were then fixed with 4% paraformaldehyde solution overnight at 4°C, washed with 70% ethanol, dehydrated (Histokinette Leica ASP2000), and then embedded in paraffin and cut into 4μm to 10μm-thick sections using a microtome (Microm HM325). For RNA and protein isolation, brain tumor tissues were snap-frozen and mechanically disrupted using stainless steel beads (69989, Qiagen) in a TissueLyser II (Qiagen).

#### RNA isolation, reverse transcription, and quantitative RT-PCR

RNA from cells and tissues was isolated with the miRNeasy Mini Kit (Qiagen) and miRNeasy Tissue/Cells Advanced Mini Kit (Qiagen), respectively. All of the procedures were performed according to the manufacturer’s instructions. A total of 500 ng of RNA was used for cDNA synthesis using the PrimeScript RT Master Mix (RR036A, TaKaRa). qRT-PCR was performed using the Rotor-Gene SYBR Green Master Mix (Qiagen).

#### Western blotting

Cells or tissues were lysed in RIPA buffer (ThermoFisher Scientific) with the addition of protease (cOmplete Mini, EDTA-free, Sigma) and phosphatase inhibitors (PhosSTOP, Sigma). Total protein extracts (20–30μg) were separated using Mini-PROTEAN precast gels and subsequently transferred onto PVDF membranes. Membranes were blocked in 5% Bovine Serum Albumin/TBST for 0.5–1 hour at room temperature and probed with primary antibodies prepared in 5%BSA/TBST overnight at 4°C. The following day, the membranes were incubated with secondary HRP-conjugated antibodies for 1 hour in room temperature and visualized with WesternBright Sirius (Advansta) using Fusion FX7. The following primary antibodies were used for immunoblotting: ARG1 (Santa Cruz Biotechnology, sc-18351), MRC1 (R&D Systems, AF2535), IL10 (R&D Systems, MAB417), ATG3 (Cell signaling, 3415), HSP90 (Santa Cruz Biotechnology, sc-13119) and GAPDH (Cell signaling, 14C10). HRP-conjugates secondary antibodies used for immunoblotting: anti-goat (DAKO, P0449), anti-rat (Cell Signaling, 7077), anti-rabbit (DAKO, P0448) and anti-mouse (DAKO, P0447).

#### BMDM isolation and polarization

To generate bone marrow-derived macrophages, femurs and tibiae of 6-week old wild-type FVBn female mice were used. Cells were flushed by centrifuging the bones cut at the knee joint. The isolate was filtered through a 40μM cell strainer and red blood cells were lysed with the ACK Lysing Buffer (ThermoFisher Scientific). Cells were plated in RPMI +10% FBS +1% Pen/Strep +50 ng/mL recombinant mouse CSF-1. BMDMs were polarized at day 7 using cytokines or by cancer cells plated into the transwell of the co-culture assay. For the M1-like polarization, 100 ng/mL LPS and 200 U/mL IFNγ were used. To obtain the M2-like phenotype, 20 ng/mL Il-4 was used. BMDMs were polarized for 24 hours and then treated with different agents (see below) for 24 hours. For the co-culture experiments, mouse glioma cells were seeded at 1 × 10^5^ cells/mL into a 0.4 μm insert a day before the polarization experiment. The next day the inserts were transferred to a 6-well plate seeded with unpolarized macrophages for 24 hours and then treated with different agents for 24 hours. The following agents were used for the treatments: imipramine (40μM, I0899 Sigma-Aldrich), desloratadine (10μM, D1069 Sigma-Aldrich), trifluoperazine (10μM, T6062 Sigma-Aldrich), HJC0197 (25μM, CAY-19092-5 Cayman Chemical), Ce3f4 (50μM, CAY-17767-10 Cayman Chemical) and histamine (50μM, HY-B1204 MedChemExpress). Small interfering RNA (siRNA) constructs were purchased from Sigma-Aldrich (MISSION predesigned siRNA; siCTRL SIC001, siHRH1 #1 SASI_Mm01_0005-8730, siHRH1 #2 SASI_Mm01_0005_8731). Cells were reverse-transfected with 25nmol of siRNA using Lipofectamine RNAiMAX (Invitrogen) in OptiMEM Reduced Serum Medium (Gibco).

#### Cell isolation and coculture experiments

Endothelial cells were isolated from the mouse tumors following the CD31 MicroBead protocol (Miltenyi Biotec, ref. 130-097-418).

CD4 and CD8 T cells were magnetically isolated from the spleen using EasySep mouse isolation kits (StemCell Technologies, ref. 19852 and 19853, respectively) according to the manufacturer’s protocol. T lymphocytes were labeled with Carboxyfluorescein Succinimidyl Ester (CFSE; Biolegend, 422701) at 1μM for 6 minutes, resuspended in the RPMI media containing 10%FBS, 1%PenStrep, NEAA and β-mercaptoethanol, and activated with the CD3/CD28 Dynabeads (ThermoFisher, 11456D). T cells were then plated at 200,000 cells/well in a 96-well plate.

CD11b cells were isolated from tumors by following the tissue harvesting and digestion protocol, as described in the sections on harvesting of mouse tissues and flow cytometry. Myeloid cells were then isolated following the protocol for the EasySep Mouse CD11b Positive Selection Kit II (StemCell Technologies, ref. 18970). After isolation, cells were plated with CD4 or CD8 T cells at a 1:1 ratio. After 72 hours of coculture, the CFSE-low T lymphocytes were stained with the live/dead cell viability reagent (LIVE/DEAD Fixable Violet Dead Cell Stain Kit, Invitrogen) for 10 minutes on ice and counted using flow cytometry (Gallios, Beckman Coulter).

#### *Ex vivo* phagocytosis assay

Tumors were harvested and digested (following the protocols described in the sections on harvesting of mouse tissues and flow cytometry), and subjected to myelin removal (Myelin Removal Beads II, human, mouse, rat, ref. 130-096-731). Cell suspensions were then stained as described in the flow cytometry protocol and sorted for CD45^+^CD11b+ and CD49d+ or CD49d- (MoFlo Astrios EQ). 20,000 FACS-sorted CD49d+/− myeloid cells were then plated in a 96-well plate, allowed to rest for 20 minutes at 37°C, spun down and resuspended in 100μL pHrodo-green *S. aureus* bioparticles (ThermoFisher, ref. P35367). After 1.5 hours of incubation, GFP high cells were counted on flow cytometry (Gallios, Beckam Coulter).

#### cAMP ELISA

Brain tumor tissues (25 mg each) were snap frozen in 0.1M HCl and homogenized with stainless steel beads in Qiagen TissueLyser II. cAMP concentrations were measured with the cAMP Direct Immunoassay Kit (ab65355, Abcam) according to the manufacturer’s protocol.

#### Histology

*For immunofluorescence*, frozen sections were dried and fixed in ice-cold methanol at −20°C for 10 minutes or fixed with 4% Paraformaldehyde for 10 minutes and then incubated in 0.1% Triton X-100 for 10 minutes to reveal intracellular proteins. Slides were then washed in PBS, blocked in 5% BSA/PBS for 30 minutes at room temperature and incubated with primary antibodies overnight at 4°C. Samples were stained with CD8 (1:200, eBioscience), FITC CD8 (1:100, Biolegend), PDGFR-β (1:100, eBioscience), Ki67 (1:100, Abcam), CD31 (1:50, Dianova), PE CD31 (1:200, Biolegend), FITC CD31 (1:200, Biolegend), CC-3 (1:100, Cell Signaling Technology), Desmin (1:200, Abcam), Alexa Fluor 647 MECA-79 (1:200, Santa Cruz Biotechnology), LAMP-1 (1:100, abcam), LC3 (1:100, Nanotools), HIF-1α (1:200, Proteintech), CXCL10 (1:200, R&D Systems), PE F4/80 (1:100, eBioscience), FITC CD45 (1:200, Biolegend), PE PD-L1 (1:100, Invitrogen), Alexa Fluor 647 CD206 (1:200, Biolegend), Alexa Fluor 647 CD8 (1:200, Biolegend) and Alexa Fluor 647 CD4 (1:200, Biolegend). The following day, sections were washed. Sections that were stained with unconjugated antibodies were incubated with the appropriate secondary fluorochrome-coupled antibodies for 1 hour at room temperature. The following secondary antibodies were used for immunofluorescence: anti-rabbit Alexa Fluor 546 (A11035, Invitrogen), anti-goat Alexa Fluor 647 (A21447, Invitrogen), and anti-rat (ab150155, Abcam). Prior to mounting with Dako fluorescence mounting medium, tissue sections were counterstained with DAPI (1:5000 dilution of 5 mg/mL stock).

*For histological assessment*, staining with hematoxylin and eosin was performed. Slides were deparaffinized with xylene, rehydrated with a graded series of ethanol and incubated in hematoxylin for 5 minutes. The samples were then incubated for few seconds in 1% acid ethanol, rinsed with water and immersed in eosin stain for 1 minute. After washing in water, tissue sections were dehydrated in ascending alcohol solutions and mounted with Eukitt Quick-hardening mounting medium (Sigma).

*For staining of mouse tissues with mouse monoclonal antibodies*, the M.O.M. kit (Vector Labs) was used. Briefly, slides were blocked with avidin/biotin, followed by blocking with the M.O.M. mouse IgG Blocking Reagent, and primary antibodies were prepared by incubation in M.O.M. diluent reagent overnight at 4°C. The next day, slides were washed, incubated in M.O.M. Biotinylated Anti-Mouse IgG Reagent, followed by incubation with fluorescent streptavidin conjugates for 45 minutes.

*For lectin staining to visualize the functional blood vasculature*, mice were intravenously injected with 100 μg/100 μL of biotinylated lectin (BK-1000, Vector Laboratories) or DyLight 594-conjugated lectin (L32471, ThermoFisher) 20 minutes before the anesthesia. Brains were then collected, dissected and processed for immunofluorescence staining.

Images were variously acquired with a Leica DM5500B fluorescent microscope, a Zeiss LSM700 UP confocal microscope, or one of two available slide scanners: a Zeiss Axioscan Z.1 and an Olympus VS120. The analysis of staining was performed with Fiji-ImageJ software or QuPath (Bankhead et al., 2017).

#### Quantification of tissue staining

Necrotic and DAPI-negative areas were excluded.

##### For total CD8^+^ T cells, CD4^+^ T cells, and CC-3+ cells

Using QuPath software, a tumor area was annotated as a region of interest. Positive cell detection was used with an intensity threshold >100. Using Fiji-ImageJ software, a Gaussian blur was applied (sigma = 2) to subtract the noise. The binary masks were created by applying a manual threshold for the positive signal (B&W). The Watershed function was utilized to avoid touching objects. The number of cells was obtained by applying ‘analyze particle’ function with a >100 pixels surface particle size.

##### For CD31^+^ area

The area of blood vessels was obtained by applying a binary mask and a manual threshold (B&W) for the CD-31 positive signal.

##### For Lectin+CD31^+^, PDGFRb+CD31^+^, and Desmin+CD31^+^ colocalization

The binary masks were created by applying a manual threshold for the positive signal (B&W). The double-positive area was determined with the Image Calculator using the ‘AND’ operator between CD31 and Lectin/PDGFRb/Desmin masks and divided by the total CD31^+^ area.

##### For CD8^+^ T cell proximity to tumor blood vessels and HIF1a-positive areas

Images were analyzed using the QuPath software (version 0.2.3) using groovy scripts. Briefly, the script (cf DistToHypoxic.groovy or DetectVesselsClassifyCellMeasureDistance.groovy) makes use of 1) cell detection in the DAPI channel with an object classifier to detect CD8^+^ cells (cf cd8+ test1.json), 2) then applying a pixel classifier to segment CD31^+^ (cf Vessel.json) or hypoxic areas (cf Hypoxic_classifier.json) and finally 3) measuring distances of cells to the nearest hypoxic area.

##### For distance of CD31-positive cells to HIF1a-positive regions

Images were analyzed using the QuPath software (version 0.2.3) using groovy scripts. Briefly, the script is makes use of 1) the pixel classifier to segment CD31^+^ areas (cf Vessel.json), 2) a pixel classifier to segment hypoxic areas (cf Hypoxic_classifier.json) and finally 3) measuring distances of CD31-positive areas to the nearest hypoxic area.

##### For LAMP1+LC3+ colocalization

Colocalization was analyzed using the JACoP plug-in for ImageJ software (Bolte and Cordelières, 2006). Threshold values were based on the single-stained and secondary antibody-only controls.

#### Flow cytometry

To obtain a single cell suspension, tumors were finely chopped, digested for 30 minutes using dispase (0.85 U/mL, Roche), collagenase A and DNAse I (144 U/mL, Roche) in DMEM-F12 medium with intermittent shaking at 37 degrees, and then passed through a 70μm cell strainer. Tumor-infiltrating leukocytes were isolated by Percoll gradient centrifugation (800xg for 45 minutes with no brake), collected at the interphase between 40% and 80% Percoll (GE Healthcare) and washed twice with FACS buffer (2%FBS/PBS). To monitor CD8 depletion, peripheral blood was collected into EDTA-coated tubes, and red blood cells were lysed using the ACK lysis buffer (Gibco). Cell pellets were washed twice in PBS and resuspended in FACS buffer. Cell suspensions were blocked with anti-CD16/32 (clone 93, Biolegend) for 10 minutes and labeled with live/dead cell viability reagent (LIVE/DEAD Fixable Red Dead Cell Stain Kit, Invitrogen) for 10 minutes on ice. For surface staining, cells were incubated with the antibodies diluted in FACS buffer on ice for 15 minutes. Fluorophore-conjugated streptavidin was used for detection of biotinylated antibodies: SA BV421 (405226, Biolegend) and SA PacO (S32365, Invitrogen). To perform intracellular immuno-staining, mice were treated with 250μg Brefeldin A for 6 hours prior to euthanasia. Cells were fixed and permeabilized with a Foxp3/Transcription Factor Staining Buffer Set (ThermoFisher Scientific) according to the manufacturer’s protocol. Intracellular staining was carried out in Perm/Wash buffer overnight at 4 degrees. After staining, cells were washed and resuspended in FACS buffer. The compensation was performed using OneComp eBeads (Invitrogen). Samples were run on a Gallios cytometer (Beckman Coulter) or a CytoFLEX S (Beckman Coulter), and all subsequent compensation and gating was performed using FlowJo software. The following antibodies were used for flow cytometry: CD45 (clone 30-F11, Biolegend), B220 (clone RA3-6B2, eBioscience), CD3 (clone 145-2C11, eBioscience), CD8 (clone 5H10, Invitrogen), CD8 (clone 53-6.7 Biolegend), IFNγ (clone XMG1.2, Biolegend), TNFα (clone MP6-XT22, Invitrogen), Ki67 (16A8, Biolegend), TCF7/TCF1 (clone S33-966, Biolegend), STAT5 (clone 47/Stat5(pY694), BD Biosciences), Granzyme B (clone NGZB, Invitrogen), Granyme B (clone QA16A02, Biolegend), CD4 (RM4-5, Biolegend), CD4 (GK1.5, Biolegend), SLAMF7 (clone #520914, R&D Systems), FoxP3 (clone FJK-16s, eBioscience), HIF-1α (clone #241812, R&D Systems), TGFβ (clone TW7-16B4, Biolegend), CD62L (clone MEL-14, Biolegend), CD44 (clone IM7, Biolegend), CD49d (clone R1-2, Biolegend), CXCL9 (clone MIG-2F5.5, Biolegend), Ly6C (clone HK1.4), Ly6G (clone 1A8, Biolegend), CD11b (clone M1/70, Biolegend), Arg1 (clone A1exF5, Invitrogen), IL10 (clone JES5-16E3, Biolegend), IL1α (clone ALF-161, Biolegend), IL6 (clone MP5-20F3, Biolegend), CD86 (clone GL-1, Biolegend), MHC-II (clone M5/114.15.2, Biolegend), and PD-L1 (clone MIH5, Invitrogen), EOMES (clone X4-83, Becton Dickinson), LAG3 (clone C9B7W, Biolegend).

#### Survival analysis in the TCGA glioblastoma dataset

We accessed the data through the UCSC Xena platform (Goldman et al., 2020). We selected the TCGA GBM cohort with AffyU133a array (n = 539).

[https://gdac.broadinstitute.org/runs/stddata__2016_01_28/data/GBM/20160128/]

The data for median were downloaded from xena.ucsc.edu and loaded to RStudio. The Kaplan Meier analyses of progression-free survival and overall survival were performed using RStudio 2022.02.01, with the package survminer version 0.4.9 (https://cran.r-project.org/web/packages/survminer/index.html).

#### Glioblastoma patient survival and antihistamine treatment

We identified glioblastoma patients treated at the University Hospital of Lausanne between June 2005 and October 2021. Patient dossiers (digitalized paper records of electronic medical records, EMRs) were validated for the diagnosis of glioblastoma. We excluded grade II tumors which transformed into higher-grade tumors and grade III brain tumors, including oligodendrogliomas and grade 3 astrocytomas. We then checked for the comedication available for each patient. We selected the antihistamine cohort of patients (n = 29). We validated that the other patients did not receive antihistamine medication and used it as the control group (n = 226). The date of diagnosis was the date of reception of the tumor material at pathology. The date of death was defined as recorded in the EMRs (censor = 1) or the last follow-up when the patient had at which the patient was alive (censor = 0).

The Kaplan Meier analysis was performed using RStudio 2022.02.01, with the package survminer version 0.4.9 (https://cran.r-project.org/web/packages/survminer/index.html).

## Data Availability

The raw data of EMRs will be made available upon request.

## Supplemental information

Document S1. Figures S1–S6 and Table S1

Document S2. Article plus supplemental information
