# Supplementary material for: Cancer cell autophagy, reprogrammed macrophages, and remodeled vasculature in glioblastoma triggers tumor immunity
Source: Cancer Cell. 2022 Oct 10;40(10):1111–1127.e9. doi: 10.1016/j.ccell.2022.08.014 (PMC9580613; doi:10.1016/j.ccell.2022.08.014)
Supplement: Document S1. Figures S1–S6 and Table S1 [file mmc1.pdf]

Cancer Cell, Volume *40*

## Supplemental information

# Cancer cell autophagy, reprogrammed macrophages, and remodeled vasculature in glioblastoma triggers tumor immunity

**Agnieszka Chryplewicz, Julie Scotton, Mélanie Tichet, Anoek Zomer, Ksenya Shchors, Johanna A. Joyce, Krisztian Homicsko, and Douglas Hanahan**

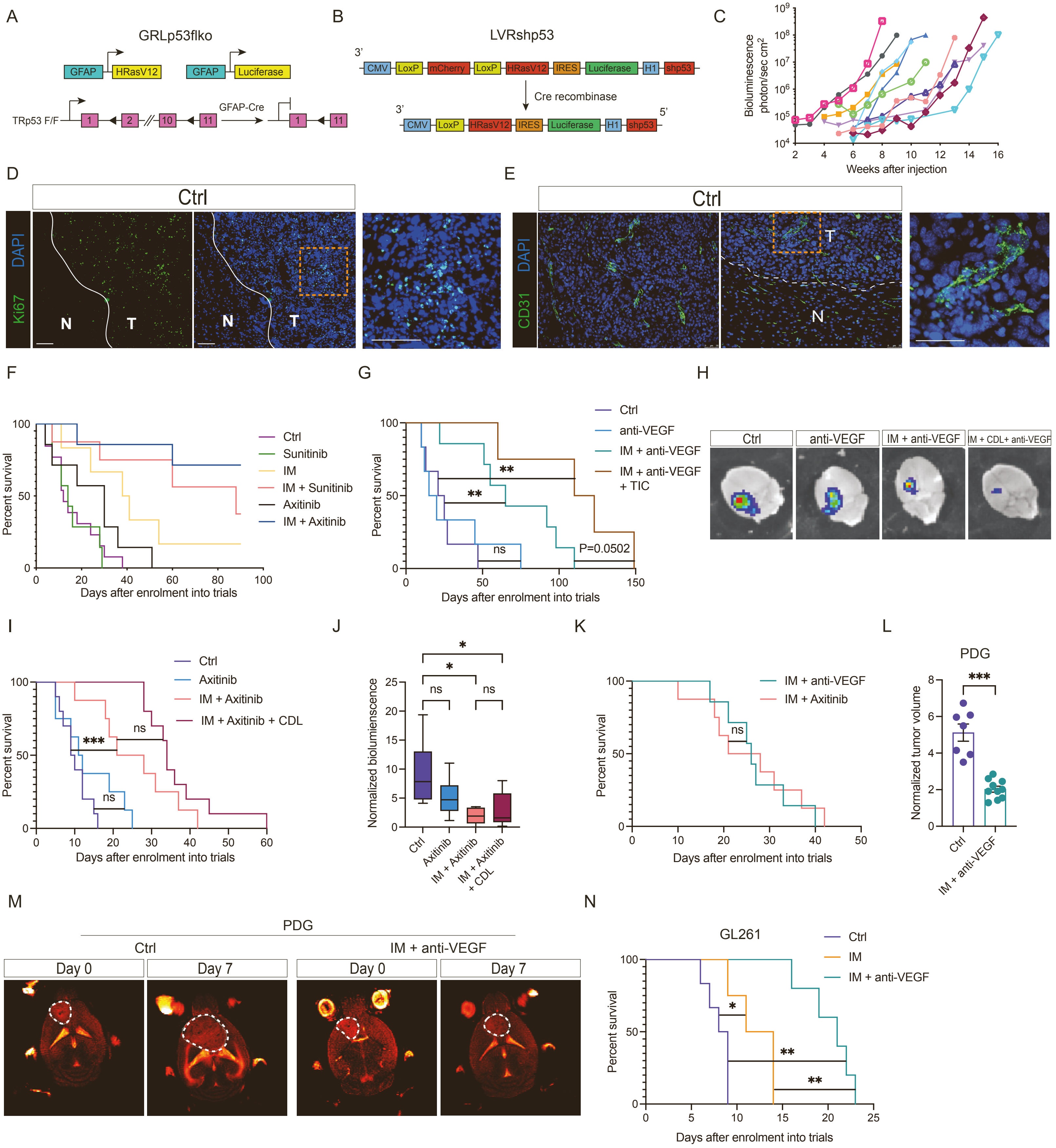

A
GRLp53flko
GFAP
HRasV12
GFAP
Luciferase
TRp53 F/F
GFAP-Cre
B
LVRshp53
CMV
LoxP
mCherry
LoxP
HRasV12
IRES
Luciferase
H1
shp53
Cre recombinase
C
Bioluminescence photon/sec cm2
Weeks after injection
D
Ctrl
DAPI
Ki67
N
T
E
Ctrl
DAPI
CD31
T
N
F
Percent survival
Days after enrolment into trials
Ctrl
Sunitinib
IM
IM + Sunitinib
Axitinib
IM + Axitinib
G
Ctrl
anti-VEGF
IM + anti-VEGF
IM + anti-VEGF + TIC
**
**
ns
P=0.0502
Percent survival
Days after enrolment into trials
H
Ctrl
anti-VEGF
IM + anti-VEGF
IM + CDL+ anti-VEGF
I
Ctrl
Axitinib
IM + Axitinib
IM + Axitinib + CDL
***
ns
ns
Percent survival
Days after enrolment into trials
J
Normalized bioluminescence
Ctrl
Axitinib
IM + Axitinib
IM + Axitinib + CDL
*
*
ns
ns
K
IM + anti-VEGF
IM + Axitinib
ns
Percent survival
Days after enrolment into trials
L
PDG
***
Normalized tumor volume
Ctrl
IM + anti-VEGF
M
PDG
Ctrl
IM + anti-VEGF
Day 0
Day 7
Day 0
Day 7
N
GL261
Ctrl
IM
IM + anti-VEGF
*
**
**
Percent survival
Days after enrolment into trials

Fig S1: **Characterization of the GBM mouse models and survival trials illustrating the survival benefit from IM + anti-VEGF therapy,** related to Fig 1

A) Diagram of the transgenes in the previously described (Shchors et al., 2015) transgenic mouse model of GBM.

B) Diagram of the pTomo HRasV12-shp53-Luc lentivirus vector, adapted from a previously described vector (Friedmann-Morvinski et al., 2016) by the addition of luciferase and shp53. The mCherry gene cassette keeps the HRasV12 gene in an off-state. When Cre recombinase deletes the mCherry cassette, HRasV12 is expressed together with luciferase and shp53.

C) Temporal evolution of bioluminescence after inoculation of the GBM-inducing lentivirus into GFAP-Cre transgenic mice. Each line represents an individual animal.

D) Ki67 (green) staining showing a high proliferative index in a representative LVRshp53 GBM tumor. T: Tumor, N: Normal adjacent brain. Scale bar 50µm. The image is representative of a whole tissue scan of 4 untreated tumors.

E) Representative images of CD31 (green) staining showing hyper- and abnormal vasculature in a LVRshp53 tumor, compared to normal adjacent tissue visible in the right panel. Scale bar 50µm. The image is illustrative of a full tumor section of 4 untreated tumors.

F) Kaplan-Meier survival analysis of tumor-bearing GRLp53het transgenic mice in cohorts treated as indicated. Ctrl (n=13), Sunitinib (n=7), IM (n=6), IM + Sunitinib (n=8), Axitinib (n=7), IM + Axitinib (n=7).

G) Survival of tumor-bearing GRLp53flko transgenic mice subjected to the indicated treatments. Ctrl (n=6), anti-VEGF (n=6), IM + anti-VEGF (n=7), IM + anti-VEGF + TIC (n=6). *p<0.05, **p<0.01 by Mantel-Cox test.

H) Illustrative ex-vivo bioluminescence imaging of brains from LVRshp53 animals subjected to the indicated treatments. Mice were enrolled in the different treatment arms with similar bioluminescence (neoplastic burden) and analyzed 1 week later. Note that in this model, single tumors typically arise, in contrast to the multi-focality of the transgenic mouse model.

I) Survival of tumor-bearing LVRshp53 animals subjected to the indicated treatments. Ctrl (n=9), Axitinib (n=8), IM + Axitinib (n=7), IM + Axitinib + CDL (n=10). ***p<0.001, ns, no statistical significance by Mantel-Cox test.

J) Normalized bioluminescence signals of LVRshp53 animals treated as indicated after 7 days. Data are represented as mean +/- SEM. Statistical analysis by one-way ANOVA with Tukey's correction. *p<0.05, ns, no statistical significance. (n=6 per group)

K) Survival of tumor-bearing LVRshp53 animals comparing the anti-VEGF Mab with a VEGFR TKI when combined with imipramine. IM + anti-VEGF (n=7), IM + Axitinib (n=7). ns, no statistical significance by Mantel-Cox test.

L) Normalized tumor volume determined by MRI in proneural PDG transgenic mice treated for 7 days with IM + anti-VEGF, compared to untreated. Ctrl (n=7), IM + anti-VEGF (n=10). ***$p<0.001$ by Mann-Whitney test. Data are presented as mean ± SEM.

M) Representative MRI images of tumors in an untreated vs. an IM + anti-VEGF treated PDG animal over the course of 7 days, from the cohorts analyzed in S1L.

N) Survival of tumor-bearing animals following orthotopic inoculation of GL261 GBM cancer cells. Ctrl (n=6), IM (n=4), IM + anti-VEGF (n=5). Statistical significance by Mantel-Cox test. *$p<0.05$, **$p<0.01$.

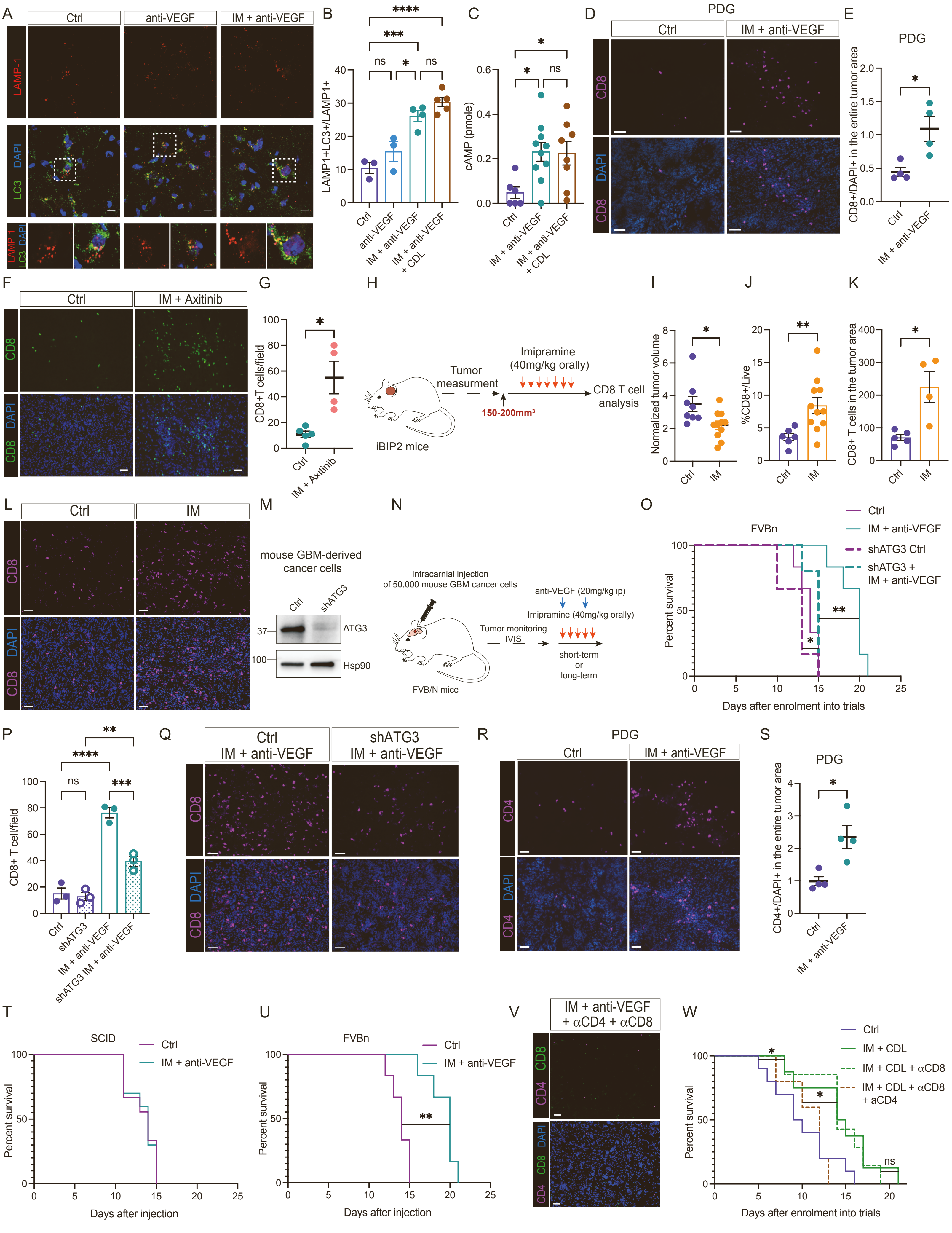

A
Ctrl
anti-VEGF
IM + anti-VEGF
LAMP-1
DAPI
LC3
B
LAMP1+LC3+/LAMP1+
Ctrl
anti-VEGF
IM + anti-VEGF
IM + anti-VEGF + CDL
C
cAMP (pmole)
D
PDG
CD8
E
CD8+/DAPI+ in the entire tumor area
F
Ctrl
IM + Axitinib
G
CD8+T cells/field
H
Tumor measurment
Imipramine (40mg/kg orally)
150-200mm³
CD8 T cell analysis
iBIP2 mice
I
Normalized tumor volume
J
%CD8+/Live
K
CD8+ T cells in the tumor area
L
IM
M
mouse GBM-derived cancer cells
shATG3
ATG3
Hsp90
N
Intracarnial injection of 50,000 mouse GBM cancer cells
anti-VEGF (20mg/kg ip)
Imipramine (40mg/kg orally)
Tumor monitoring IVIS
short-term or long-term
FVB/N mice
O
FVBn
Ctrl
IM + anti-VEGF
shATG3 Ctrl
shATG3 + IM + anti-VEGF
Percent survival
Days after enrolment into trials
P
CD8+ T cell/field
shATG3 IM + anti-VEGF
Q
Ctrl IM + anti-VEGF
shATG3 IM + anti-VEGF
R
CD4
S
CD4+/DAPI+ in the entire tumor area
T
SCID
Days after injection
U
FVBn
V
IM + anti-VEGF + αCD4 + αCD8
W
IM + CDL
IM + CDL + αCD8
IM + CDL + αCD8 + aCD4

Fig S2: **T lymphocytes are recruited by IM + anti-VEGF in multiple mouse models, and induction of autophagy is functionally important for anti-tumor immunity,** related to Fig 1

A) Representative images of LC3 (green) and LAMP-1 (red) immunostaining of LVRshp53 tumor samples subjected to the indicated treatment for one week. Bottom panels illustrate the co-localization indicative of the culmination of autophagy in lysosomes, magnified 2.5X. Scale bar 10µm. Images are illustrative of the analysis shown in Fig S2B.

B) Quantification of LC3 and LAMP1 co-localization as a percentage of total LAMP1 positive lysosomes in LVRshp53 tumor samples subjected to the indicated treatment for one week. Bars represent mean +/- SEM. Value for each replicate was obtained by averaging the quantification for 6-9 fields per tumor, each from a different mouse. Ctrl (n=3), anti-VEGF (n=3), IM + anti-VEGF (n=4), IM + anti-VEGF + CDL (n=5). Statistical analysis by one-way ANOVA with Tukey's correction. ****$p<0.0001$, ***$p<0.001$, ns, no statistical significance.

C) cAMP levels in control tumors and tumors treated with IM + anti-VEGF or IM + anti-VEGF + CDL, adjusted to tumor weight. Ctrl (n=6), IM + anti-VEGF (n=10), IM + anti-VEGF + CDL (n=8). Statistical analysis by one-way ANOVA with Tukey's correction. Bars represent mean +/- SEM. *$p<0.05$, ns, no statistical significance.

D) Representative images of CD8 (magenta) and DAPI nuclear staining (blue) of proneural PDG tumors treated as indicated for 1 0 days. Scale bar 50µm. Images represent the analysis shown in Fig S2E.

E) Quantification of CD8 T cells in control (n=4) and IM + anti-VEGF (n=4) tumors from the proneural PDG model. Each dot indicates the total number of CD8 T cells in the entire cross-sectional area of a tissue section from one tumor. Statistical analysis by Mann-Whitney test. *$p<0.05$. Data are presented as mean ± SEM.

F) Representative images of CD8 (green) and DAPI nuclear staining (blue) of LVRshp53 tumors treated with IM plus the VEGFR inhibitor Axitinib, as quantified in Fig S2G. Scale bar 50µm.

G) Quantification of CD8 T cells in LVRshp53 tumors treated with IM plus the VEGFR inhibitor axitinib. Each dot represents the average of 8 to 14 images for one mouse. Ctrl (n=5), IM + Axitinib (n=4). Statistical analysis by Mann-Whitney test. *$p<0.05$. Data are presented as mean ± SEM.

H) Schematic of the short-term trials for testing the efficacy of IM in the iBIP2 mouse model of BRAF-driven melanoma.

I) Normalized iBIP2 tumor volume after 7 days. Ctrl (n=8), IM (n=11). Statistical analysis by Mann-Whitney test. *$p<0.05$. Data are presented as mean ± SEM.

J) Flow cytometry analysis of CD8 T cells in the live cell compartment of iBIP2 melanoma tumors treated as indicated. Ctrl (n=6), IM (n=11). **$p<0.01$ by Mann-Whitney test. Data are presented as mean ± SEM.

K) Quantification of CD8 immunostaining as a percentage of DAPI-positive cells in full tissue sections of iBIP2 tumors. Ctrl (n=5), IM (n=4). *$p<0.05$ by Mann-Whitney test. Data are presented as mean ± SEM. Data are presented as mean ± SEM.

L) Representative images of CD8 (magenta) and nuclei (DAPI, blue) in untreated and IM-treated melanoma tumors. Images are illustrative of the analysis shown in Fig S2K. Scale bar 50µm.

M) Western blot analysis of ATG3 in mouse glioma-derived control and shATG3-transfected cells in culture.

N) Schematic of the orthotopic syngeneic mouse model designed to evaluate CD8 T cell influx upon ATG3 knockdown in cancer cells.

O) Survival of mice following orthotopic inoculation with control or shATG3 glioma cell lines treated with IM + anti-VEGF. Ctrl (n=6), IM + anti-VEGF (n=6), shATG3 Ctrl (n=6), shATG3 IM + anti-VEGF (n=5). Statistical analysis by Mantel-Cox test. *$p<0.05$, **$p<0.01$.

P) Quantification of CD8 T cells in control and shATG3 tumors treated with IM + anti-VEGF. Each dot indicates the average of 7 to 11 images from one tumor; tumors from 3 mice were analyzed for each condition. Statistical analysis by one-way ANOVA with Tukey's correction. **$p<0.01$, ***$p<0.001$, ****$p<0.0001$. Data are presented as mean ± SEM.

Q) Representative images of CD8 (magenta) and nuclei (DAPI, blue) in control and shATG3 tumors treated with IM + anti-VEGF. Images are illustrative of the analysis presented in Fig S2P. Scale bar 50µm.

R) CD4 (magenta) and DAPI nuclear staining (blue) of proneural PDG tumors treated with IM + anti-VEGF for 10 days, representative of the analysis shown in Fig S2S. Scale bar 50µm.

S) Quantification of CD4 T cells in control (n=4) and IM + anti-VEGF (n=4) tumors from the PDG model. Each dot indicates the total number of CD8 T cells in the entire cross-sectional area of a tissue section from one tumor; 4 tumors were analyzed, each from a different mouse. Statistical analysis by Mann-Whitney test. *$p<0.05$. Data are mean ± SEM.

T) Survival of immunocompromised SCID animals orthotopically transplanted with a mouse-derived glioma cell line and treated with IM + anti-VEGF. Experiments from panel T and U were performed at the same time with the same batch of cells. Ctrl (n=9) and IM + anti-VEGF (n=10). Ns, no statistical significance by Mantel-Cox test.

U) Survival of immunocompetent FVBn animals orthotopically transplanted with a mouse-derived glioma cell line and treated with IM + anti-VEGF. Ctrl (n=6) and IM + anti-VEGF (n=6). ** $p<0.01$ by Mantel-Cox test.

V) Representative image from a tumor-bearing LVRshp53 animal treated with IM + CDL plus depleting antibodies to CD8 and CD4 T cells, illustrating the depletion. Image is illustrative of a whole-tumor scan from 3 different tumors. Scale bar 50μm.

W) Survival of tumor-bearing LVRshp53 animals treated with IM + CDL +/- depleting antibodies to assess the functional contributions of CD8 and CD4 T cells to survival benefit. Ctrl (n=10), IM + CDL (8), IM + CDL + αCD8 + αCD4 (n=5), IM + CDL + αCD8 (n=7). * $p<0.05$, ns, no statistical significance by Mantel-Cox test.

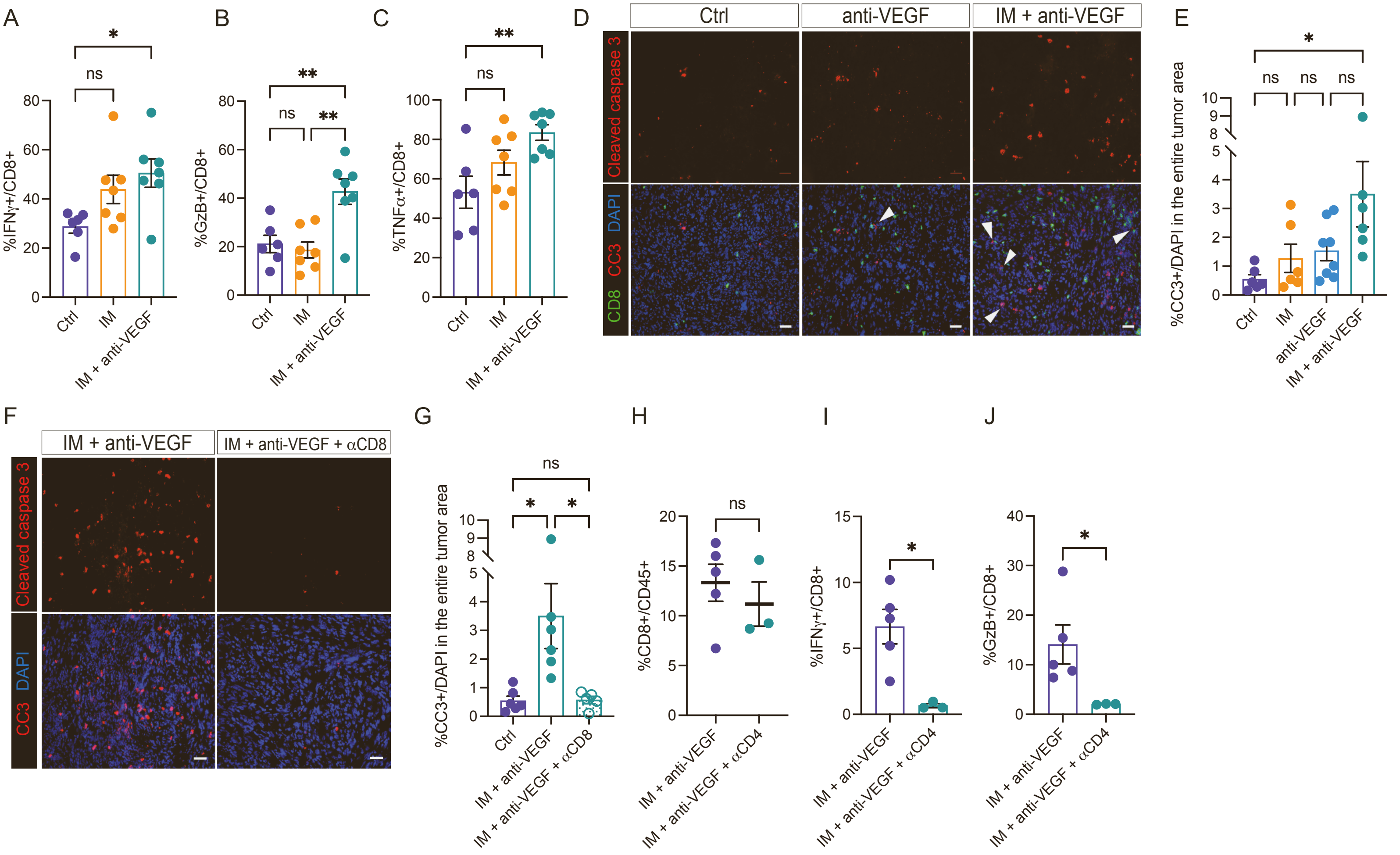

A
%IFNγ+/CD8+
Ctrl
IM
IM + anti-VEGF
ns
*
B
%GzB+/CD8+
ns
**
**
C
%TNFα+/CD8+
ns
**
D
Ctrl
anti-VEGF
IM + anti-VEGF
Cleaved caspase 3
CD8 CC3 DAPI
E
%CC3+/DAPI in the entire tumor area
ns
ns
ns
*
F
IM + anti-VEGF
IM + anti-VEGF + αCD8
Cleaved caspase 3
CC3 DAPI
G
%CC3+/DAPI in the entire tumor area
IM + anti-VEGF + αCD8
ns
*
*
H
%CD8+/CD45+
IM + anti-VEGF + αCD4
ns
I
%IFNγ+/CD8+
*
J
%GzB+/CD8+
*

Fig S3: **Further characterization of the activities of intratumoral CD8 and CD4 T cells,** related to Fig 2

A) Flow cytometry analysis of IFNγ intracellular staining in fixed and permeabilized CD8+T cells isolated from tumors treated with IM or IM + anti-VEGF. Ctrl (n=6), IM (n=7), IM + anti-VEGF (n=7). Statistical analysis by one-way ANOVA with Tukey's correction. *p<0.05, ns, no statistical significance.
B) Flow cytometry analysis of GzB intracellular staining in fixed and permeabilized CD8 T cells, as in A). Ctrl (n=6), IM (n=7), IM + anti-VEGF (n=7). Statistical analysis by one-way ANOVA with Tukey's correction. **p<0.01, ns, no statistical significance.
C) Flow cytometry analysis of TNFα intracellular staining in fixed and permeabilized CD8 T cells, as in A). Ctrl (n=6), IM (n=7), IM + anti-VEGF (n=7). Statistical analysis by one-way ANOVA with Tukey's correction. **p<0.01, ns, no statistical significance.
D) Representative Cleaved Caspase-3 (CC-3) (red), CD8 (green) and DAPI (blue) staining of LVRshp53 tumors subjected to indicated treatments. Images are representative of 8-10 images from 3 different tumors. White arrows show adjacent CD8+ and CC-3+ cells. Scale bar 50μm.
E) Quantification of CC-3 positive cells in the entire tumor area in LVRshp53 tumors. Ctrl (n=6), IM (n=6), anti-VEGF (n=8), IM + anti-VEGF (n=6). *p<0.05, ns, no statistical significance by one-way ANOVA with Tukey`s correction.
F) Representative CC-3 (red) and DAPI staining of CD8-depleted and nondepleted LVRshp53 tumors undergoing treatment with IM + anti-VEGF. Images are illustrative of 8-10 images from 3 different tumors. Scale bar 50μm.
G) Quantification of CC-3 immunostaining as a percentage of DAPI-positive cells in the whole LVRshp53 tumor area. Ctrl (n=6), IM + anti-VEGF (n=6), IM + anti-VEGF + αCD8 (n=5). Statistical analysis by one-way ANOVA. *p<0.05, ns, no statistical significance.
H) Flow cytometry analysis of CD8 T cells in tumors treated with IM + anti-VEGF +/- depletion of CD4 T cells. IM + anti-VEGF (n=5), IM + anti-VEGF + αCD4 (n=3). Statistical analysis by Mann-Whitney test, ns, no statistical significance.
I) Flow cytometry analysis of IFNγ intracellular staining in fixed and permeabilized CD8 T cells from tumors treated with IM + anti-VEGF +/- depletion of CD4 T cells. IM + anti-VEGF (n=5), IM + anti-VEGF + αCD4 (n=3). Statistical analysis by Mann-Whitney test, *p<0.05.

J) Flow cytometry analysis of GzB intracellular staining in fixed and permeabilized CD8 T cells, as for H/I). IM + anti-VEGF (n=5), IM + anti-VEGF + αCD4 (n=3). *$p<0.05$ by Mann-Whitney test.

Quantitative data in all relevant panels are presented as mean ± SEM.

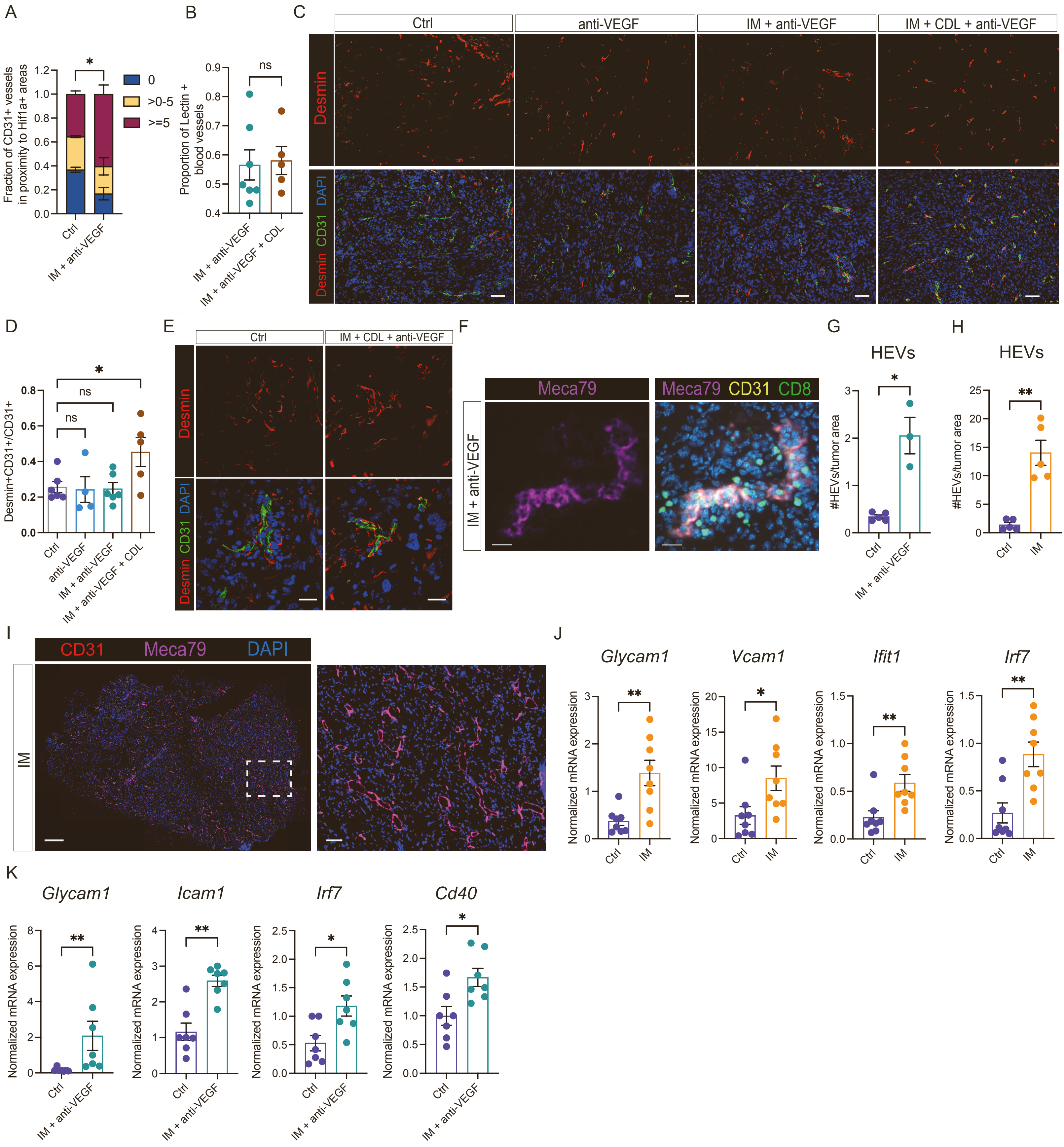

A
Fraction of CD31+ vessels in proximity to Hif1a+ areas
0
>0-5
>=5
Ctrl
IM + anti-VEGF
B
Proportion of Lectin + blood vessels
ns
IM + anti-VEGF
IM + anti-VEGF + CDL
C
Ctrl
anti-VEGF
IM + anti-VEGF
IM + CDL + anti-VEGF
Desmin
Desmin CD31 DAPI
D
Desmin+CD31+/CD31+
ns
ns
Ctrl
anti-VEGF
IM + anti-VEGF
IM + anti-VEGF + CDL
E
Ctrl
IM + CDL + anti-VEGF
Desmin
Desmin CD31 DAPI
F
Meca79
Meca79 CD31 CD8
IM + anti-VEGF
G
HEVs
#HEVs/tumor area
Ctrl
IM + anti-VEGF
H
HEVs
#HEVs/tumor area
Ctrl
IM
I
CD31 Meca79 DAPI
IM
J
Glycam1
Vcam1
Ifit1
Irf7
Normalized mRNA expression
Ctrl
IM
K
Glycam1
Icam1
Irf7
Cd40
Normalized mRNA expression
Ctrl
IM + anti-VEGF

Fig S4: **Tumor vessels are remodeled ('quasi-normalized') upon treatment with IM + anti-VEGF in GBM and melanoma tumors,** related to Fig 3

A) Fraction of CD31+ vessels located in HIF1α+ zones in the entire area of full sections of GBM tumors. The zones were divided into 0μm (i.e., within the Hif1a+ zone), >0 and <5μm, and >5μm. Ctrl (n=5), IM + anti-VEGF (n=6). *p<0.05 by Mann-Whitney test.

B) The proportion lectin+ blood vessels is not affected by the addition of CDL. Each dot indicates the average of 8 to 12 fields in tissue sections from a GBM tumor from one mouse. IM + anti-VEGF (n=7), IM + anti-VEGF + CDL (n=5). Statistical analysis by Mann Whitney test. Bars represent mean +/- SEM. ns, no statistical significance.

C) Representative immunofluorescence of desmin (red), CD31 (green) and DAPI (blue) in animals treated for 1 week, as indicated. Images are representative of the analysis performed in Fig S4C. Scale bar 50μm.

D) Quantification of desmin+ and CD31+ cells as a percentage of CD31 positive areas within tissue sections from tumors treated as indicated. Ctrl (n=6), anti-VEGF (n=4), IM + anti-VEGF (n=6), IM + anti-VEGF + CDL (n=5). Statistical analysis by one-way ANOVA. *p<0.05, ns, no statistical significance.

E) Representative high magnification images of desmin+ and CD31+ cells. Scale bar 10μm. Images are representative of the analysis performed in Fig S4C.

F) Representative immunofluorescence of MECA79 (magenta), CD31 (bright yellow) and CD8 (green) of proneural PDG tumors treated as indicated for 10 days. Scale bar 20μm. Images are illustrative of the analysis in Fig S4E.

G) Quantification of immunostaining for HEVs in PDG tumors treated with IM + anti-VEGF (n=3) or untreated controls (n=5). The data are shown as number of HEVs per $mm^2$ of tumor tissue. *p<0.05 by Mann-Whitney test.

H) Quantification of immunostaining for HEVs in iBIP2 melanoma tumors treated with IM. Ctrl (n=5) and IM (n=5). The data are shown as number of HEVs per $mm^2$ of tumor tissue. **p<0.01 by Mann-Whitney test.

I) Representative immunofluorescence images of MECA79 (magenta), CD31 (red) and DAPI (blue) in iBIP2 melanoma tumors treated with IM. Scale bars 250μm and 50μm. Images are illustrative of the analysis shown in Fig S4F.

J) mRNA expression of *Glycam1, Vcam1, Ifit1* and *Irf7* in CD31+ endothelial cells from melanoma tumors subjected to IM (n=8) or untreated (n=8). Statistical significance by Mann-Whitney test. *p<0.05, **p<0.01.

K) mRNA profiling of endothelial cells isolated from PDG glioma tumors. The expression of *Glycam1, Icam1, Irf7* and *Cd40* was normalized to *18S* RNA. $*p<0.05$, $**p<0.01$ by Mann-Whitney test.

Quantitative data in all relevant panels are presented as mean ± SEM.

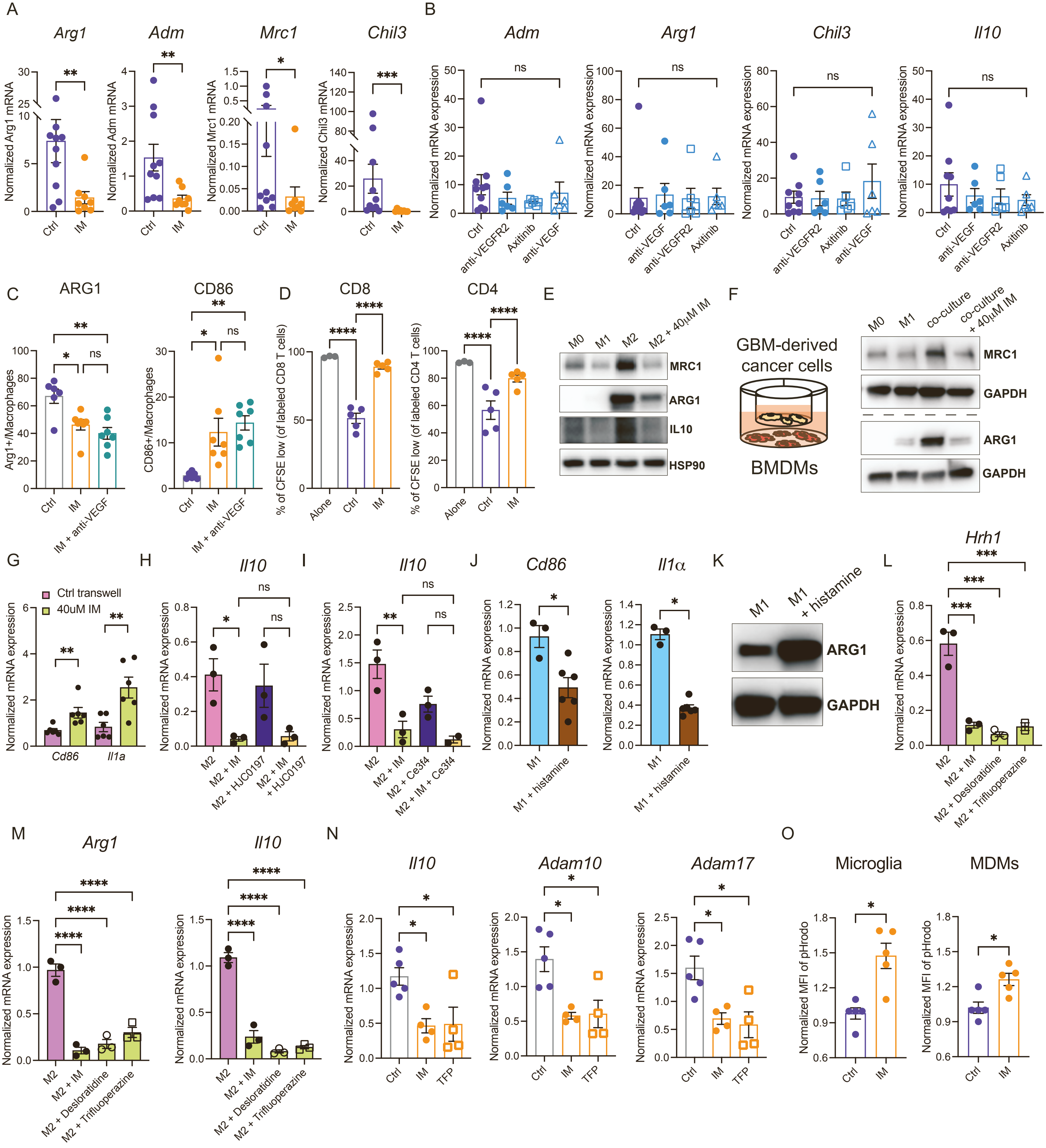

A
Arg1
Adm
Mrc1
Chil3
B
Adm
Arg1
Chil3
Il10
Ctrl
IM
anti-VEGFR2
Axitinib
anti-VEGF
Normalized mRNA expression
C
ARG1
CD86
Arg1+/Macrophages
CD86+/Macrophages
IM + anti-VEGF
D
CD8
CD4
% of CFSE low (of labeled CD8 T cells)
% of CFSE low (of labeled CD4 T cells)
Alone
E
M0
M1
M2
M2 + 40μM IM
MRC1
ARG1
IL10
HSP90
F
GBM-derived cancer cells
BMDMs
co-culture
co-culture + 40μM IM
GAPDH
G
Ctrl transwell
40uM IM
Cd86
Il1a
H
Il10
M2 + IM
M2 + HJC0197
M2 + IM + HJC0197
I
M2 + Ce3f4
M2 + IM + Ce3f4
J
Cd86
Il1α
M1 + histamine
K
M1 + histamine
L
Hrh1
M2 + Desloratidine
M2 + Trifluoperazine
M
N
Adam10
Adam17
TFP
O
Microglia
MDMs
Normalized MFI of pHrodo

Fig S5: **Imipramine reprograms immunosuppressive myeloid cells via HRH1 targeting,** related to Fig 4

A) mRNA expression of the M2-like macrophage markers *Arg1, Adm, Mrc1 and Chil3* in Ctrl (n=10) and IM-treated (n=8) LVRshp53 tumors. ***p<0.001, **p<0.01, *p<0.05 by Mann-Whitney test.

B) mRNA expression of the M2-like markers *Adm, Arg1, Chil3* and *Il10* in Ctrl (n=9-10), anti-VEGFR2 (DC101) (n=6), Axitinib (n=6), anti-VEGF (n=6) treated tumors. ns, no statistical significance by one-way ANOVA.

C) Flow cytometry analysis of ARG1 and CD86 expression in proneural PDG glioma tumors treated for one week. Ctrl (n=6), IM (n=7), IM + anti-VEGF (n=7). Macrophages were gated as CD45+CD11b+Ly6C-Ly6G-. Statistical analysis by one-way ANOVA with Tukey's correction. *p<0.05, **p<0.01, ns, no statistical significance.

D) Co-cultures of tumoral CD11b cells from PDG tumors combined with activated splenic CSFE-labeled CD8 or CD4 T cells. Each dot represents the average of 1-3 technical replicates. T cells alone (n=3), Ctrl co-cultures (n=5), and co-cultures with anti-VEGF (n=3) or IM (n=5). Statistical analysis by two-way ANOVA. ****p<0.0001, ns, no statistical significance.

E) Western blot analysis of ARG1, MRC1 and IL10 in *ex-vivo* polarized bone marrow-derived macrophages. M2-like BMDMs were treated with 40μM imipramine for 24 hours. Experiment was repeated 3 times.

F) Western blot analysis of MRC1 and ARG1 in polarized bone marrow-derived macrophages in transwell co-cultures with cancer cells derived from the LVRshp53 GBM mouse model. M2-like BMDMs were treated with 40μm imipramine for 24 hours. Data are representative of 2 independent experiments.

G) Analysis of the M1-like marker genes *CD86* and *IL1α* in polarized BMDMs in a transwell assay with cancer cells +/- IM as assessed by qRT-PCR and normalized to *18S* RNA. Cells were treated with 40μm imipramine for 24 hours. **p<0.01 by Mann-Whitney test. Data are representative of 2 independent experiments.

H) Analysis of *Il10* expression in M2-polarized BMDMs treated with 40μM IM, 25 μM HJC0197 or the combination for 24 hours. Expression is normalized to *18S* RNA. *p<0.05, ns, no statistical significance by one-way ANOVA. Data are representative of 2 independent experiments.

I) Analysis of *Il10* expression in M2-polarized BMDMs treated with 40μM IM, 50 μM Ce3f4 or the combination for 24 hours. Expression is normalized to *18S* RNA. *p<0.05, ns, no statistical significance by one-way ANOVA. Data are representative of 2 independent experiments.

J) mRNA levels of *Cd86* and *Il1a* in M1-polarized BMDMs treated with 50μM histamine for 24 hours. *p<0.05 by Mann-Whitney test. Data are representative of 2 independent experiments.

K) Western blot analysis of ARG1 in one of the replicates from panel J).

L) mRNA expression of *Hrh1* normalized to *18S* RNA in M2-polarized BMDMS, either untreated or treated with 40μm imipramine, 10μm desloratadine or 10μm trifluoperazine for 24 hours. ***p<0.001 by one-way ANOVA with Tukey's correction. Data are representative of 2 independent experiments.

M) Analysis of the *Arg1* and *Il10* expression in M2-polarized macrophages treated as indicated, as assessed by qRT-PCR. Cells were treated with 40 μm imipramine, 10 μm desloratadine or 10 μm trifluoperazine for 24 hours. ****p<0.0001 by one-way ANOVA with Tukey's correction. Data are representative of 2 independent experiments.

N) mRNA expression of *Il10, Adam10* and *Adam17* in CD11b cells isolated from tumors treated with IM (n=4) or TFP (n=4) or untreated controls (n=5). Statistical analysis by one-way ANOVA. *p<0.05.

O) Sorted microglia and MDMs from untreated or IM-treated PDG glioma tumors assayed with green pHrodo Staphylococcus aureus bioparticles to assess their capability for phagocytosis. Data presented as MFI of pHrodo/live cells. Statistical analysis by Mann-Whitney test. *p<0.05.

Quantitative data in all relevant panels are presented as mean ± SEM.

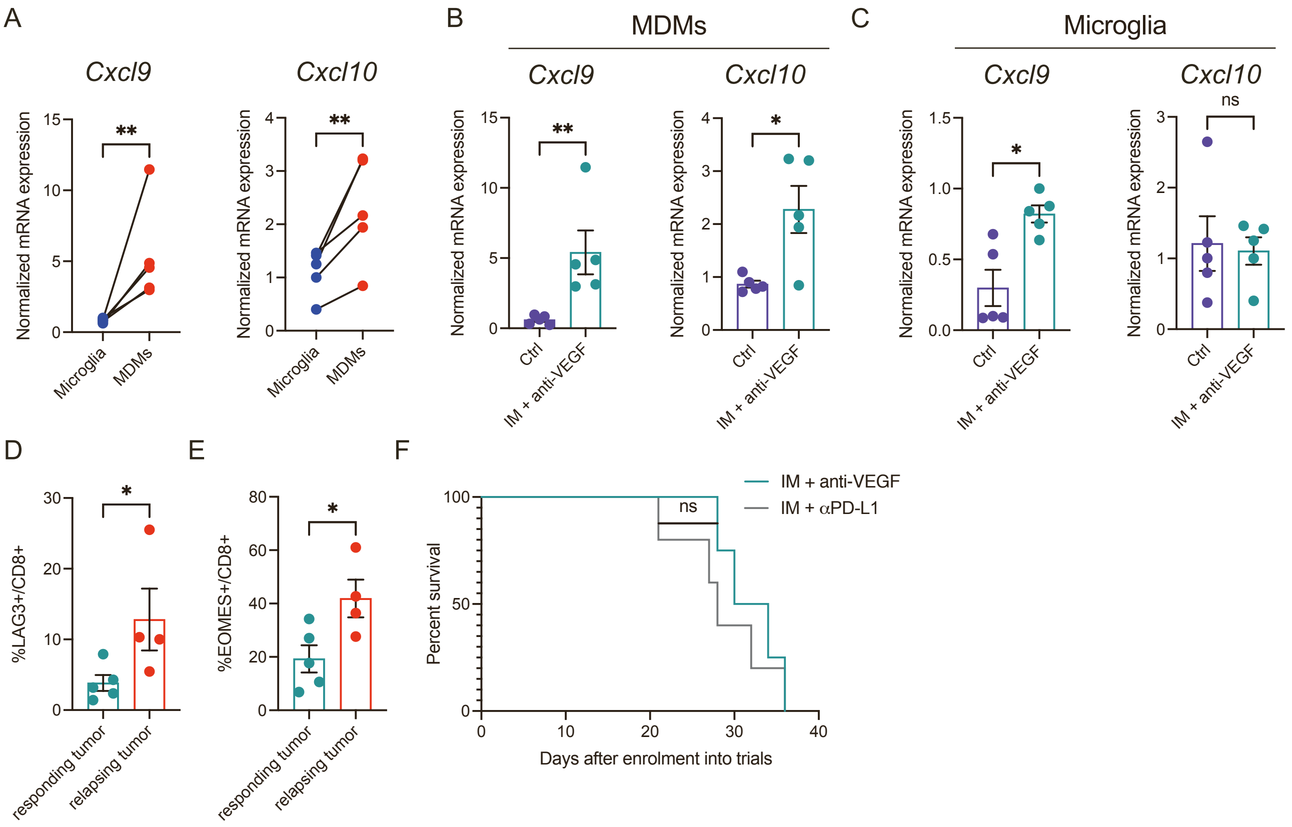
A
Cxcl9
Cxcl10
Normalized mRNA expression
**
Microglia
MDMs
B
MDMs
Cxcl9
Cxcl10
Ctrl
IM + anti-VEGF
C
Microglia
ns
D
%LAG3+/CD8+
*
responding tumor
relapsing tumor
E
%EOMES+/CD8+
F
IM + anti-VEGF
IM + αPD-L1
Percent survival
Days after enrolment into trials

Fig S6: **CXCR3 ligands are also upregulated in proneural (PDG) tumors in response to IM + anti-VEGF, and the combination of IM + αPD-L1 has a similar therapeutic benefit to IM + anti-VEGF in LVRshp53 animals,** related to Fig 6 and Fig 7

A) mRNA expression of *Cxcl9* and *Cxcl10* in sorted microglia and MDMs from proneural glioma tumors in PDG transgenic mice treated with IM + anti-VEGF (n=5). Statistical analysis by ratio paired t test. $^{**}p<0.01$.

B) mRNA expression of *Cxcl9* and *Cxcl10* in untreated (n=5) or IM + anti-VEGF-treated (n=5) FACS-sorted MDMs from PDG tumors as assessed by qRT-PCR. Statistical analysis by Mann-Whitney test. $^{*}p<0.05$, $^{**}p<0.01$.

C) mRNA expression of *Cxcl9* and *Cxcl10* in untreated (n=5) or IM + anti-VEGF-treated (n=5) FACS-sorted microglia from PDG tumors as assessed by qRT-PCR. $^{*}p<0.05$, ns, no statistical significance by Mann-Whitney test.

D) Flow cytometry analysis of LAG3 staining in CD8 T cells in responding (n=5) and relapsing tumors (n=4). Statistical analysis by Mann-Whitney test. $^{*}p<0.05$.

E) EOMES expression in CD8 T cells assessed by flow cytometry. Responding tumors (n=5), relapsing tumors (n=4). $^{*}p<0.05$ by Mann-Whitney test.

F) Survival of tumor-bearing LVRshp53 animals subjected to IM + anti-VEGF (n=5) or IM + αPD-L1 (n=4). Statistical significance by Mantel-Cox test. ns, no statistical significance.

Quantitative data in all relevant panels are presented as mean ± SEM."

Table S1: **List of primers used for qRT-PCR**, related to STAR Methods section RNA isolation, reverse transcription, and quantitative RT-PCR

| Name | Forward sequence | Reverse sequence |
|---|---|---|
| *18s* | GTAACCCGTTGAACCCCATT | CCATCCAATCGGTAGTAGCG |
| *Gapdh* | AGGTCGGTGTGAACGGAT | TGTAGACCATGTAGTTGAGGTCA |
| *Adm* | CACCCTGATGTTATTGGG | TTAGCGCCCACTTATTCCACT |
| *Arg1* | CAGAAGAATGGAAGAGTCAG | CAGATATGCAGGGAGTCACC |
| *Chil3* | CAGGTCTGGCAATTCTTCTGA | GTCTTGCTCATGTGTGTAAGTGA |
| *Mrc1* | CTCTGTTCAGCTATTGGACGC | CGGAATTTCTGGGATTCAGCTTC |
| *Il10* | GCTCTTACTGACTGGCATGAG | CGCAGCTCTAGGAGCATGTG |
| *iNos* | TTTGCTTCCATGCTAATGCGAAAG | GCTCTGTTGAGGTCTAAAGGCTCCG |
| *F13a1* | GAGCAGTCCCGCCCAATAAC | CCCTCTGCGGACAATCAACTTA |
| *Il1α* | GCACCTTACACCTACCAGAGT | AAACTTCTGCCTGACGAGCTT |
| *Cd86* | TGTTTCCGTGGAGACGCA | TTGAGCCTTTGTAAATGG |
| *Hrh1* | CTGGTGCTGTATGCAGTGC | GCTACCGACAGGCTGACAA |
| *Stab1* | GGCAGACGGTACGGTCTAAAC | AGCGGCAGTCCAGAAGTATCT |
| *Il1r2* | GTTTCTGCTTTCACCACTCCA | GAGTCCAATTTACTCCAGGTCAG |
| *Cxcl9* | GGAGTTCGAGGAACCCTAGTG | GGGATTTGTAGTGGATCGTGC |
| *Cd49d* | GATGCTGTTGTTGTACTTCGG | ACCACTGAGGCATTAGAGAGC |
| *Glycam1* | TCAGCTGCAACCACCTCAG | TTCGTGATACGACTGGCACC |
| *Icam1* | GTGATGCTCAGGTATCCA | CACAGTTCTCAAAGCACA |
| *Vcam1* | AGTTGGGGATTCGGTTGTTCT | CCCCTCATTCCTTACCACCC |
| *Irf7* | GAGACTGGCTATTGGGGGAG | GACCGAAATGCTTCCAGGG |
| *Cd40* | TTGTTGACAGCGGTCCATCTA | GCCATCGTGGAGGTACTGTTT |
| *Ifit1* | CTGAGATGTCACTTCACATGGAA | GTGCATCCCCAATGGGTTCT |
| *Mmp2* | CAAGTTCCCCGGCGATGTC | TTCTGGTCAAGGTCACCTGTC |
| *Mmp9* | CTGGACAGCCAGACACTAAAG | CTCGCGGCAAGTCTTCAGAG |
| *Adam10* | ATGGTGTTGCCGACAGTGTTA | GTTTGGCACGCTGGTGTTTTT |

| | | |
|---|---|---|
| *Adam17* | AGGACGTAATTGAGCGATTTTGG | TGTTATCTGCCAGAAACTTCCC |
